# Supplementary material for: Investigation of the Role of Myocyte Orientations in Cardiac Arrhythmia Using Image-Based Models
Source: Biophys J. 2019 Oct 8;117(12):2396–408. doi: 10.1016/j.bpj.2019.09.041 (PMC6990390; doi:10.1016/j.bpj.2019.09.041)
Supplement: Document S1. Figs. S1–S7 and Tables S1–S3 [file mmc1.pdf]

Biophysical Journal, Volume *117*

Supplemental Information

Investigation of the Role of Myocyte Orientations in Cardiac Arrhythmia Using Image-Based Models

Dominic G. Whittaker, Alan P. Benson, Irvin Teh, Jürgen E. Schneider, and Michael A. Colman

1. Supplementary Figures

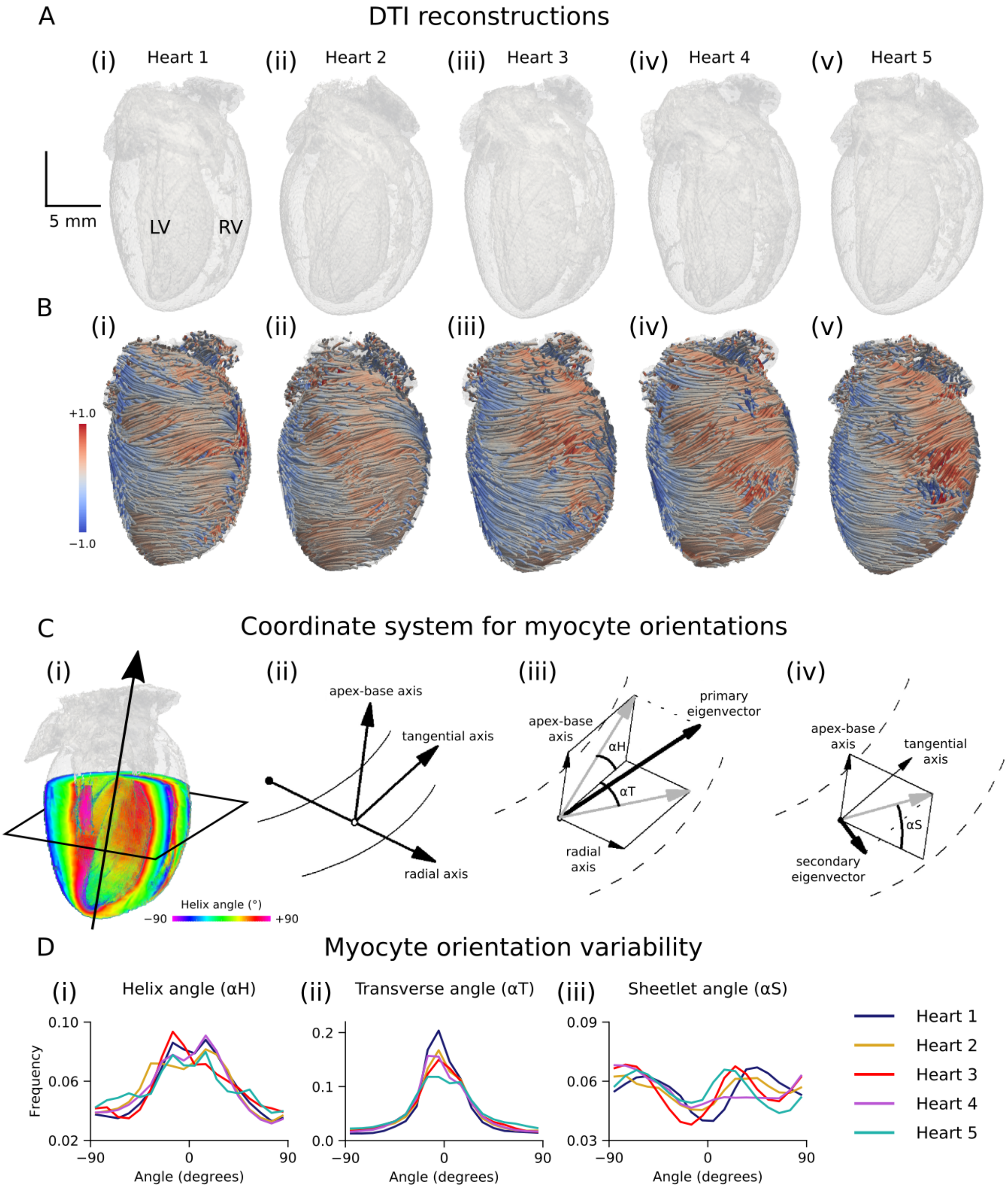

Figure S1: Anatomical variability from DTI reconstructions and co-ordinate system used to quantify myocyte organisation. (A) Five healthy rat heart reconstructions from *ex vivo* DTI (1), and (B) streamlines coloured according to the *z* component of the myocyte orientation. (C) Co-ordinate system used to compute myocyte and sheetlet orientation angles.

(i) For each heart a base-apex axis is fitted to the centre of the left ventricle, normal to the transverse plane of the heart (shown as a rectangle in the short-axis plane). (ii) Three orthogonal reference axes are defined for each voxel, from which (iii) the helix angle (αH) and transverse angle (αT) are calculated from the primary eigenvector, and (iv) sheetlet angle (αS) is calculated from the secondary eigenvector (2). (D) Myocyte orientation variability between the five ventricular geometries – (i) helix angle, (ii) transverse angle, and (iii) sheetlet angle.

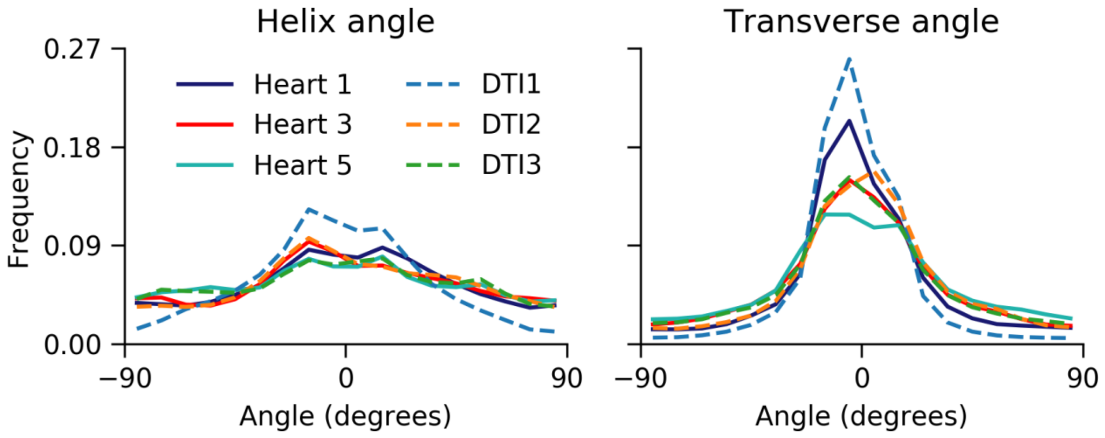

Figure S2: Myocyte orientation variability in the processed DTI datasets. Variability between the three DTI-based myocyte orientation scenarios as quantified by the helix angle (left panel) and transverse angle (right panel), compared with the unprocessed DTI data from Hearts 1, 3, and 5.

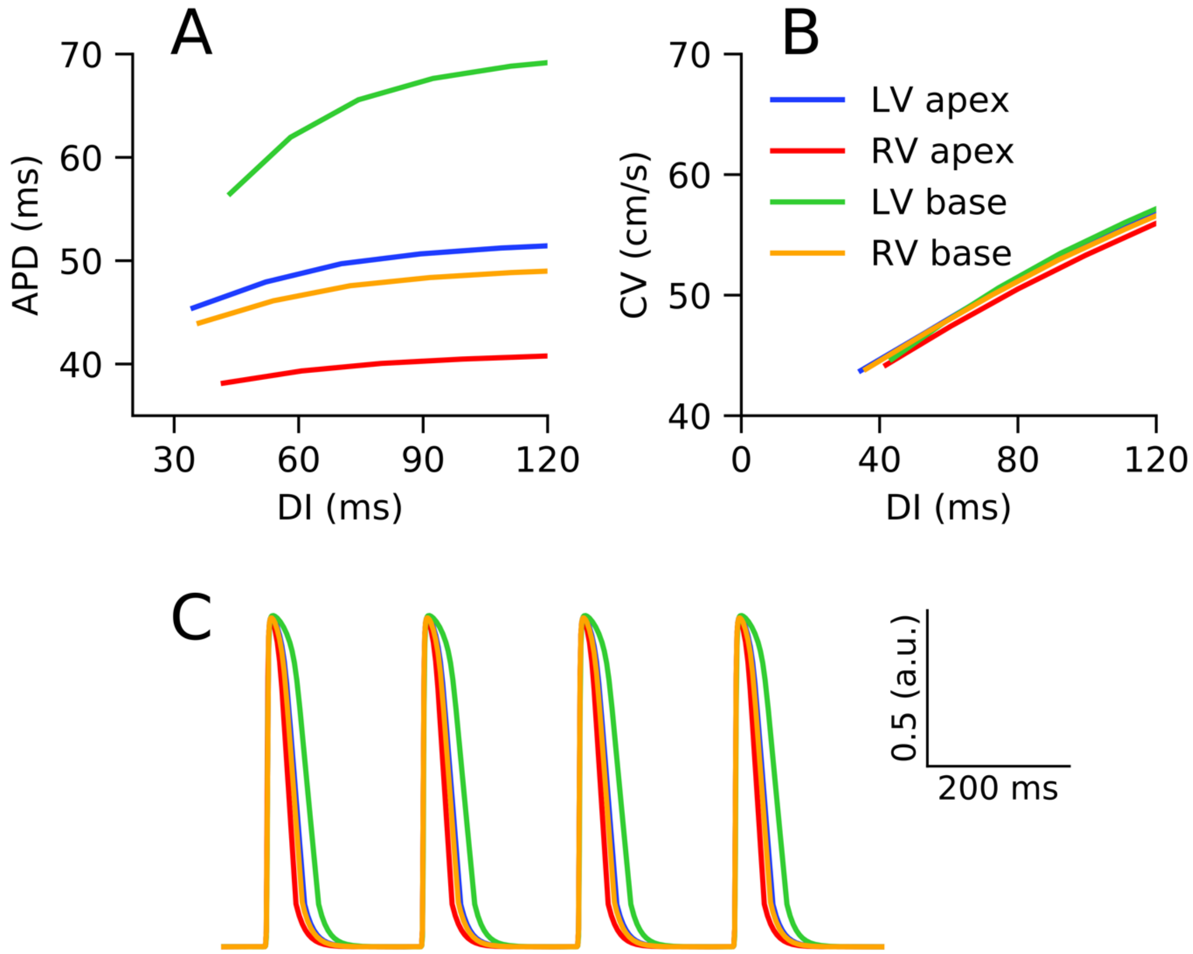

Figure S3: Electrophysiological heterogeneity in Protocol 2. (A) Action potential duration and (B) conduction velocity restitution curves for different regions of the ventricles, used in Protocol 2. (C) A train of rat ventricular cell action potentials using the modified FK3V regional cell models at a pacing rate of 5 Hz. Regional cell models were created by adjusting the τ_r parameter in the updated FK3V model by ±20% in order to reproduce experimental differences in rat ventricular APD (3).

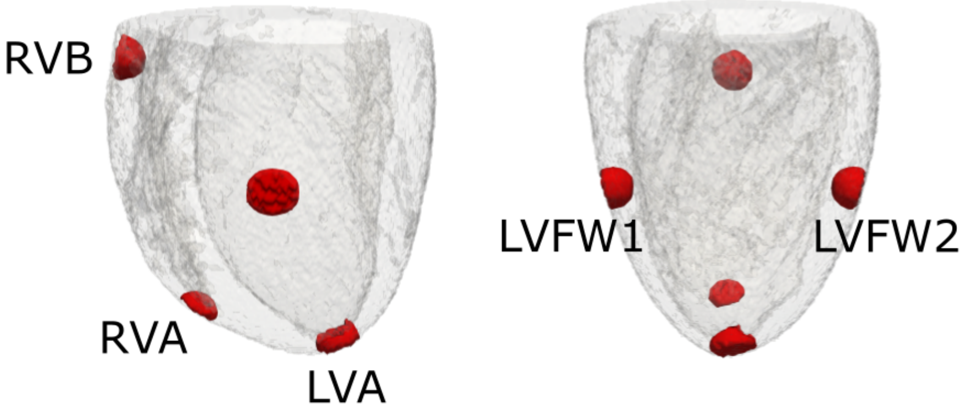

Figure S4: Stimulus locations for Protocol 2. The bi-ventricular geometry used in this study (shown in transparent grey), with stimulus locations used in Protocol 2 highlighted in red. Locations are as follows: right ventricular base (RVB); right and left ventricular apex (RVA and LVA, respectively); left ventricular free wall location 1 and 2 (LVFW1 and LVFW2, respectively).

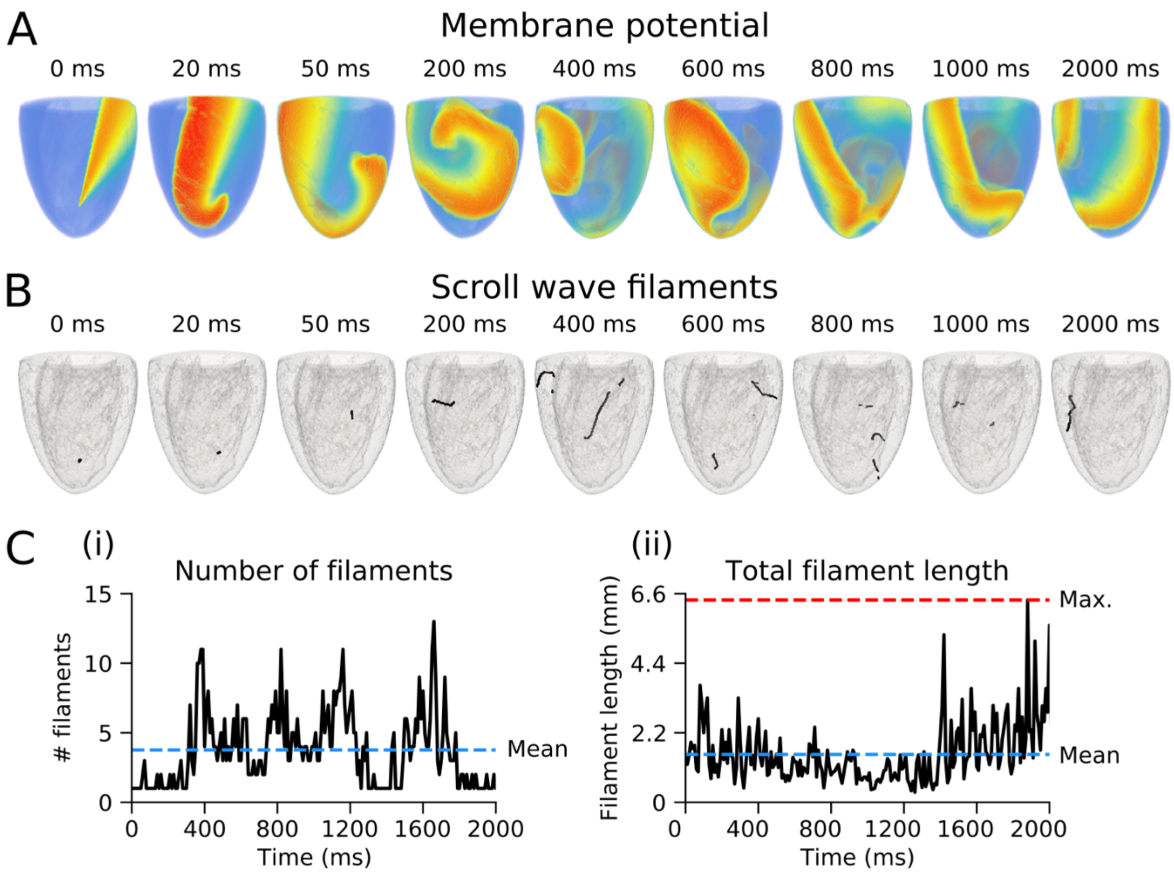

Figure S5: Arrhythmia initiation and scroll wave filaments. (A) Initiation and evolution of scroll waves on the left ventricular lateral wall using the phase distribution method (4). (B) Corresponding scroll wave filaments shown as black lines on a semi-transparent contour of the geometry. (C) Time series of (i) the number of filaments over a 2000 ms period (mean is shown with a blue dotted line), and (ii) the total filament length (mean and max. shown with blue and red dotted lines, respectively).

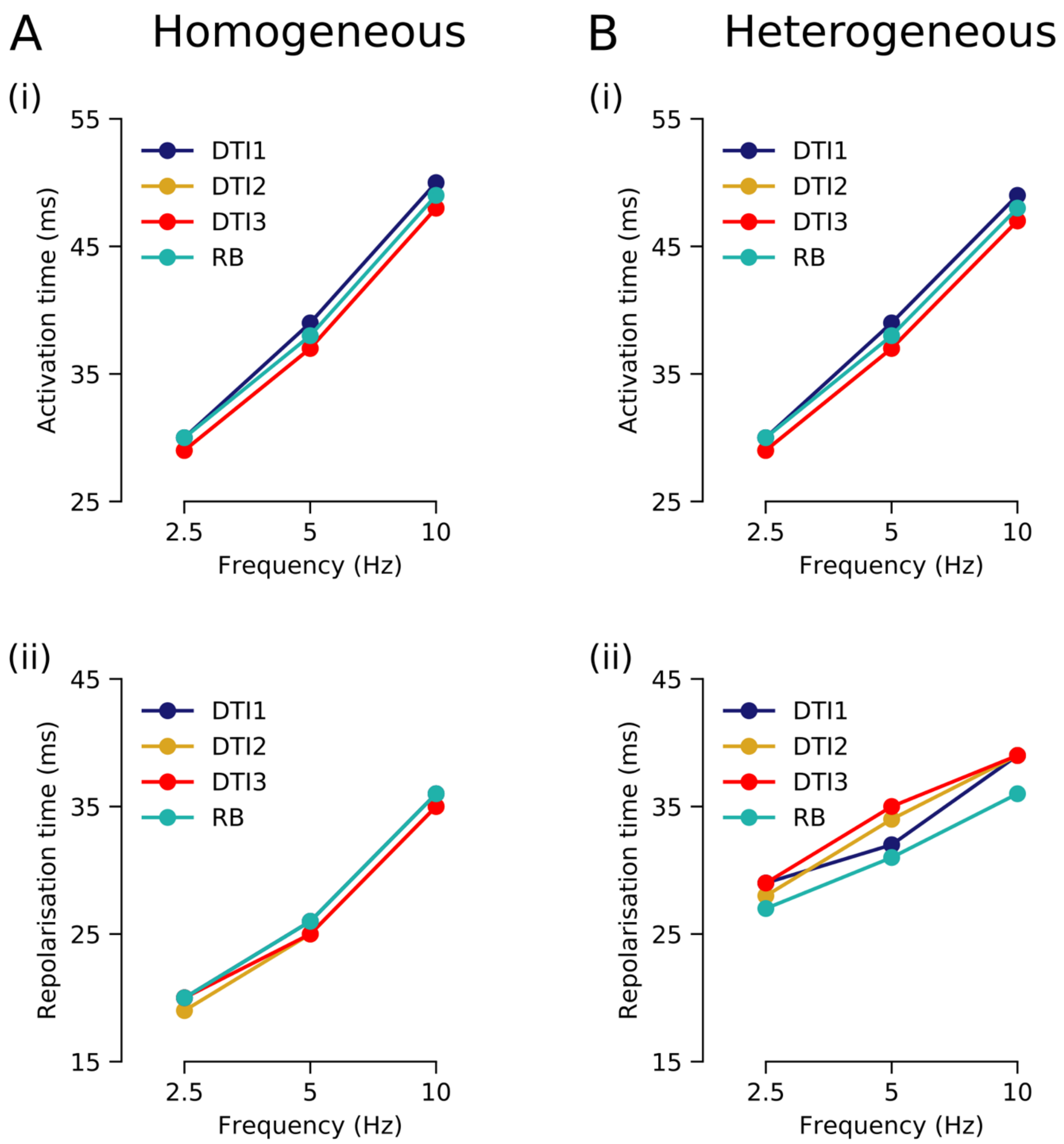

Figure S6: Effects of myocyte orientations on activation and repolarisation times. A summary of total (i) activation and (ii) repolarisation times for all myocyte orientation scenarios under (A) homogeneous and (B) heterogeneous conditions at pacing rates of 2.5, 5, and 10 Hz (corresponding to cycle lengths of 400, 200, and 100 ms, respectively).

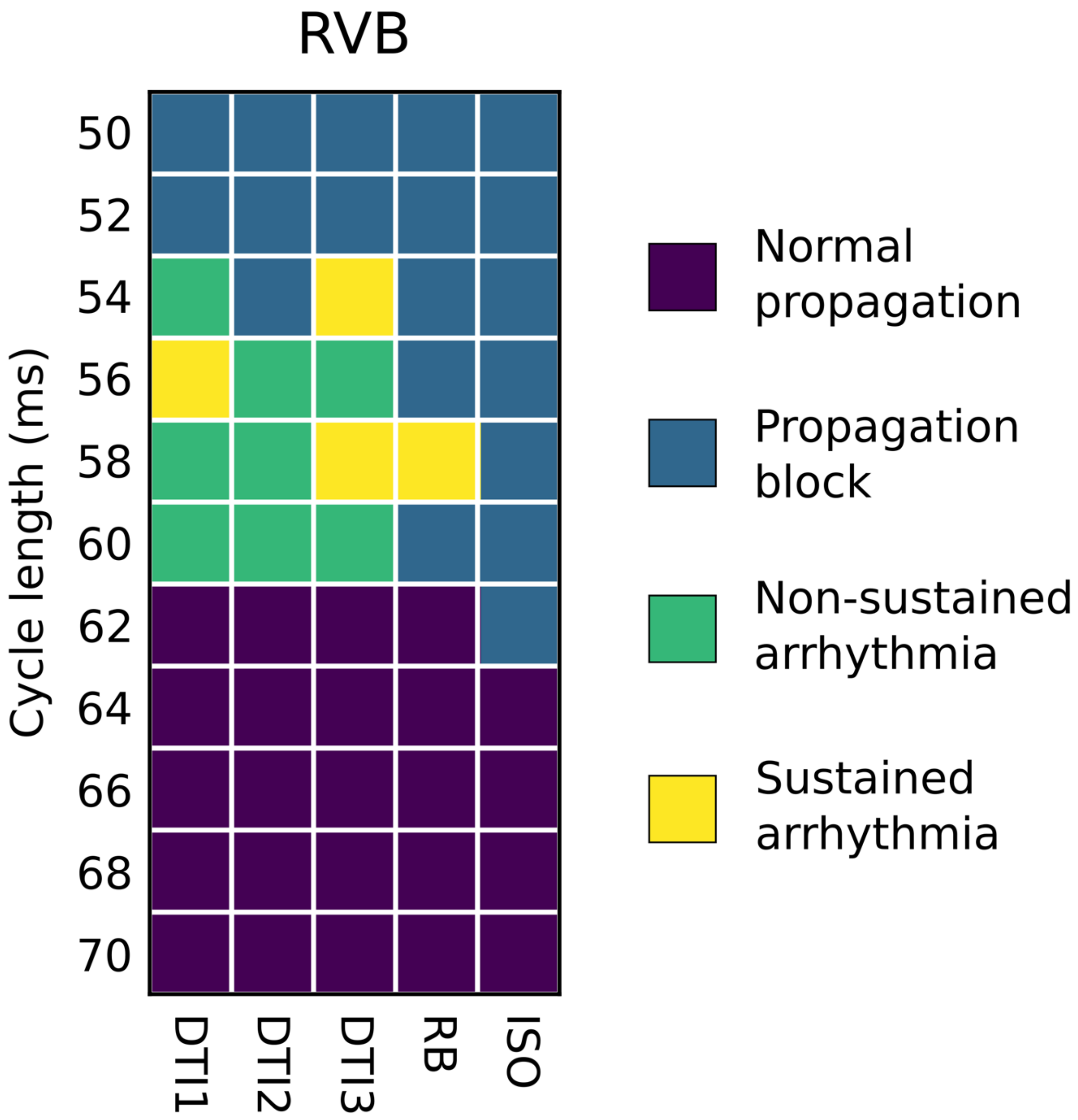

Figure S7: Comparison of arrhythmia inducibility under anisotropic and isotropic conditions. Vulnerability grids for arrhythmia inducibility following rapid pacing across a range of cycle lengths at the right ventricular base (RVB) under the four myocyte orientation scenarios and under isotropic (ISO) conditions.

3. Supplementary Tables

Supplementary Table S1: A summary of data from (1, 5) **used in this study.**

	**Heart 1**	**Heart 2**	**Heart 3**	**Heart 4**	**Heart 5**
**Total volume (mL)**	0.556	0.627	0.546	0.609	0.553
**Myocyte orientations in this study**	DTI1		DTI2		DTI3

A summary of the data used to create the hybrid bi-ventricular geometry and DTI-based myocyte orientation scenarios used in this study. Healthy rat heart reconstructions from DTI at 100 µm resolution (1) were previously cropped into bi-ventricular geometries (5), with the total volumes shown in the table. The three geometries with the closest total volumes (1, 3, and 5) were merged to form a single bi-ventricular geometry with the microstructure from Hearts 1, 3, and 5 forming the basis of myocyte orientation scenarios DTI1, DTI2, and DTI3, respectively.

Supplementary Table S2: A summary of arrhythmia inducibility for Protocol 2.

	RV apex	RVB	LVFW1	LVFW2	LV apex	Total
**DTI1**	× (0)	✓ (4)	✓ (1)	× (0)	× (0)	5
**DTI2**	✓ (2)	✓ (3)	✓ (1)	× (0)	✓ (3)	9
**DTI3**	✓ (1)	✓ (4)	✓ (1)	× (0)	✓ (2)	8
**RB**	✓ (1)	✓ (1)	✓ (2)	✓ (1)	✓ (1)	6

A summary of whether or not an arrhythmia was inducible for DTI-based (DTI1, DTI2, and DTI3) and rule-based (RB) myocyte orientations for five locations – right ventricular (RV) apex, RV base, left ventricular free wall 1 and 2 (LVFW1 and LVFW2, respectively), and LV apex, as well as a total across all five locations. Boxes marked with a cross denote that no arrhythmias were inducible, whereas boxes marked with a tick denote that an arrhythmia was inducible (the number of pacing rates tested at which re-entry could be induced is given in brackets).

Supplementary Table S3: ***p*** **values for one-way ANOVA.**

	p **value**
**Mean number of filaments**	0.569
**Mean total filament length**	0.395
**Max. total filament length**	0.521

A summary of p values from one-way ANOVA for the mean number of filaments, mean total filament length, and maximum total filament length from $n = 10$ re-entry simulations.

4. Supplementary Videos

Video S1: Effects of myocyte orientation variability on ventricular activation. A cross-sectional view into the left and right ventricular cavities showing activation over a 100 ms period under different myocyte orientation scenarios at a pacing rate of 5 Hz. Excited tissue is red whereas recovered tissue is blue.

Video S2: Effects of myocyte orientation variability on arrhythmia induction. A right ventricular anterior wall view of rapid pacing at a cycle length of 60 ms at the right ventricular apex under different myocyte orientation scenarios, shown over a period of 1000 ms. Excited tissue is red whereas recovered tissue is blue.

Video S3: Effects of myocyte orientation variability on arrhythmia dynamics. A left ventricular anterior wall view of the dynamic evolution of re-entrant waves and corresponding scroll wave filaments over a 1000 ms period. Filaments are shown as black lines on a semi-transparent contour of the geometry. Excited tissue is red whereas recovered tissue is blue.

Video S4: A mechanism of re-entry induction in Protocol 2 due to myocyte organisation. A bi-ventricular wedge view of wave propagation (excited tissue shown in red on a semi-transparent contour of the geometry) and myocyte orientation streamlines for the DTI1 (left) and DTI2 (right) scenarios, following left ventricular free wall stimulation (LVFW1) in Protocol 2. For DTI2 the initial stimulus wave propagates preferentially along the vertical axis compared to more horizontally for DTI1. The second stimulus captures and travels towards the RV (a region of lower APD) where wave breakthrough occurs for DTI1, whereas

for DTI2 the wave travels more along the direction of apico-basal heterogeneity, and so meets the longer APD of the base. In both cases the APD is longer in the LV than RV, so the wave gets blocked as it tries to propagates away from the RV (to the right in the video). This critical difference leads to the fourth and fifth (but not third) stimuli being captured for DTI1, compared to the third and fifth stimuli for DTI2. The repolarisation heterogeneity induced by multiple rapid activations causes the fifth stimulus to generate a wave which breaks for DTI1, causing even more repolarisation asymmetry. This ultimately causes the wave from the sixth stimulus to break and degenerate into re-entry for DTI1, but not DTI2.

References

1. Teh, I., D. McClymont, R.A.B. Burton, M.L. Maguire, H.J. Whittington, C.A. Lygate, P. Kohl, and J.E. Schneider. 2016. Resolving Fine Cardiac Structures in Rats with High-Resolution Diffusion Tensor Imaging. Sci. Rep. 6: 30573.

2. Benson, A.P., O. Bernus, H. Dierckx, S.H. Gilbert, J.P. Greenwood, A.V. Holden, K. Mohee, S. Plein, A. Radjenovic, M.E. Ries, G.L. Smith, S. Sourbron, and R.D. Walton. 2011. Construction and validation of anisotropic and orthotropic ventricular geometries for quantitative predictive cardiac electrophysiology. Interface Focus. 1: 101–116.

3. Watanabe, T., L.M.D. Delbridge, J.O. Bustamante, and T.F. Mcdonald. 1983. Heterogeneity of the action potential in isolated rat ventricular myocytes and tissue. Circ. Res. 52: 280–290.

4. Biktashev, V.N., and A.V. Holden. 1998. Reentrant waves and their elimination in a model of mammalian ventricular tissue. Chaos Interdiscip. J. Nonlinear Sci. 8: 48–56.

5. Whittaker, D.G., A.P. Benson, I. Teh, J.E. Schneider, and M.A. Colman. 2018. Role of cardiac microstructure variability on ventricular arrhythmogenesis. In: 2018 Computing in Cardiology Conference (CinC). .
