# Supplementary material for: Investigation of the Role of Myocyte Orientations in Cardiac Arrhythmia Using Image-Based Models
Source: Biophys J. 2019 Oct 8;117(12):2396–408. doi: 10.1016/j.bpj.2019.09.041 (PMC6990390; doi:10.1016/j.bpj.2019.09.041)
Supplement: Document S2. Article plus Supporting Material [file mmc6.pdf]

Biophysical Journal
Article

Biophysical Society

Investigation of the Role of Myocyte Orientations in Cardiac Arrhythmia Using Image-Based Models

Dominic G. Whittaker,[1,2] Alan P. Benson,[1] Irvin Teh,[3] Jürgen E. Schneider,[3] and Michael A. Colman[1,*]
[1]School of Biomedical Sciences, Faculty of Biological Sciences, University of Leeds, Leeds, United Kingdom; [2]Centre for Mathematical Medicine & Biology, School of Mathematical Sciences, University of Nottingham, Nottingham, United Kingdom; and [3]Experimental and Preclinical Imaging Centre, Leeds Institute of Cardiovascular and Metabolic Medicine, University of Leeds, Leeds, United Kingdom

ABSTRACT Cardiac electrical excitation-propagation is influenced by myocyte orientations (cellular organization). Quantitatively understanding this relationship presents a significant research challenge, especially during arrhythmias in which excitation patterns become complex. Tissue-scale simulations of cardiac electrophysiology, incorporating both dynamic action potential behavior and image-based myocardial architecture, provide an approach to investigate three-dimensional (3D) propagation of excitation waves in the heart. In this study, we aimed to assess the importance of natural variation in myocyte orientations on cardiac arrhythmogenesis using 3D tissue electrophysiology simulations. Three anatomical models (i.e., describing myocyte orientations) of healthy rat ventricles—obtained using diffusion tensor imaging at 100 μm resolution—were registered to a single biventricular geometry (i.e., a single cardiac shape), in which the myocyte orientations could be represented by each of the diffusion tensor imaging data sets or by an idealized rule-based description. The Fenton-Karma cellular excitation model was modified to reproduce rat ventricular action potential duration restitution to create reaction-diffusion cardiac electrophysiology models. Over 250 3D simulations were performed to investigate the effects of myocyte orientations on the following: 1) ventricular activation, 2) location-dependent arrhythmia induction via rapid pacing, and 3) dynamics of re-entry averaged over multiple episodes. It was shown that 1) myocyte orientation differences manifested themselves in local activation times, but the influence on total activation time was small; 2) differences in myocyte orientations could critically affect the inducibility and persistence of arrhythmias for specific stimulus-location/cycle-length combinations; and 3) myocyte orientations alone could be an important determinant of scroll wave break, although no significant differences were observed in averaged arrhythmia dynamics between the four myocyte orientation scenarios considered. Our results show that myocyte orientations are an important determinant of arrhythmia inducibility, persistence, and scroll wave break. These findings suggest that where specificity is desired (for example, when predicting location-dependent, patient-specific arrhythmia inducibility), subject-specific myocyte orientations may be important.

SIGNIFICANCE The pumping of the heart is coordinated by the rhythmic propagation of electrical impulses. Irregular rhythms, or "arrhythmias," occur when electrical activity becomes complex and overrides normal pacemaking, leading to loss of cardiac output and, often, sudden death. Conduction patterns in the heart are critically influenced by cellular "myocyte" orientations, yet the extent to which variation between subjects facilitates the development of arrhythmias remains unresolved and is often overlooked. Using novel, high-resolution, image-based computational models of the mammalian heart incorporating gross anatomy and myocyte orientations, we show that differences in myocyte organization alone between subjects critically influences inducibility and the persistence of arrhythmias. Our findings signify that myocyte orientations are an important consideration for arrhythmia inducibility risk quantification on a patient-specific basis.

INTRODUCTION

Cardiac arrhythmias, including ventricular tachycardia (VT) and ventricular fibrillation (VF), are leading causes of morbidity and mortality globally, yet remain incompletely understood (1). Arrhythmias are complex, multiscale phenomena dependent on cellular electrophysiology (i.e., ion currents and homeostasis) yet emerging only in tissue, in which electrical propagation is influenced by structural features at both the macroscopic and microscopic scales (pertaining to gross anatomy, including wall thickness and valve openings and cellular organization and connections,

Submitted April 16, 2019, and accepted for publication September 23, 2019.
*Correspondence: m.a.colman@leeds.ac.uk
Editor: Zhilin Qu.
https://doi.org/10.1016/j.bpj.2019.09.041
© 2019 Biophysical Society.
This is an open access article under the CC BY license (http://creativecommons.org/licenses/by/4.0/).

which determine conduction pathways, respectively). A detailed understanding of the interacting role of all of these features is vital for a full dissection of arrhythmia mechanisms and ideally requires direct visualization of excitation-propagation within the three-dimensional (3D) in vivo beating heart (2). This is not yet technically feasible, and studying arrhythmias continues to present a significant research challenge.

Mapping electrical activity during arrhythmias, using techniques such as optical imaging, is one approach that has been used to shed light on the mechanisms of VF (3,4). Reaction-diffusion models of cardiac electrophysiology, which incorporate both dynamic action potential (AP) behavior and myocardial architecture from imaging modalities such as diffusion tensor imaging (DTI) (5) and contrast-enhanced micro-CT (6), offer an alternative approach for studying the complex 3D organization of excitation waves during arrhythmias (2,7). An integrative computational approach offers the advantage of allowing the investigator direct control of important factors that influence arrhythmia dynamics, such as ionic current properties and tissue structure and anisotropy (8). Furthermore, studying the filament dynamics of scroll waves offers an approach to quantify the complex spatiotemporal activity that underlies arrhythmias such as VF (9).

Mathematical modeling of cardiac electrophysiology has proven a useful tool for unraveling mechanisms of ventricular arrhythmogenesis, including under pathophysiological conditions and pharmacological modulation (10–14). However, the field of computational cardiac modeling, which is now more than half a century old (15), is beginning to move beyond the single virtual heart paradigm as the need to account for intersubject variability has begun to emerge (16). Recent studies have focused in particular on cellular electrophysiological variability, with an emphasis on how this might influence proarrhythmic risk and pharmacological response (17). The effects of intersubject variability in the complex myocyte orientations of the heart (i.e., cellular organization, typically termed "fiber orientation" in the literature) on arrhythmia dynamics, however, have been less extensively studied; such analysis would therefore provide an important insight into arrhythmia mechanisms and patient variability.

A standard approximation in simulation studies, in the absence of high-resolution sample-specific information, is to assign myocyte orientations to models of the heart using a set of rules (18,19) representing a "one size fits all" approach. As computational cardiac models are being used increasingly for safety-critical applications such as arrhythmia risk quantification on a patient-specific basis (20), however, there is also a growing need to understand how important these features are for the specificity of reproducing electrical patterns in individual patients. We sought to characterize quantitatively how variability in myocyte orientations influences ventricular activation and arrhythmia dynamics. Specifically, we aimed to assess if myocyte organization is an important factor determining intersubject variability in the following: 1) activation patterns and total activation time; 2) specific arrhythmia dynamics; and 3) overall vulnerability to arrhythmia; and therefore, its importance for predictive patient-specific models. To this end, we created a single bi-ventricular geometry in which everything but the myocyte orientations was fixed, eliminating all other non-structural determinants of electrical activity. It was hypothesized that intersubject variability in cardiac myocyte orientations plays an important role in determining ventricular arrhythmia dynamics.

MATERIALS AND METHODS

DTI reconstructions

Diffusion tensor magnetic resonance imaging (MRI) represents the state-of-the-art in nondestructive determination of myocyte orientations in the heart (21). This method is based on characterization of the diffusivity of water molecules throughout the myocardium, which probe the tissue microstructure through revealing preferential directions of diffusion at the microscopic scale (22). Previously, five healthy rat hearts were perfusion and immersion fixed ex vivo using low osmolality Karnovsky's fixative and then imaged using a diffusion-weighted fast spin echo sequence at a resolution of 100 μm isotropic (23). Myocyte and sheetlet orientation angles were subsequently reconstructed from these data sets using methods described in detail elsewhere (23,24). Ventricular geometries were segmented out, and variability in ventricular myocyte orientations (helix, transverse, and sheetlet angles) were quantified, as shown in Fig. S1. It can be seen from histograms of the angles that whereas the overall morphology of the distributions was consistent (e.g., a peak in the transverse angle at ~0°), a degree of variability in the organization of myocyte and sheetlet orientation angles existed between the five data sets (for a more detailed quantitative characterization, see (23)).

In this study, three of these hearts with the most similar shapes and volumes (Hearts 1, 3, and 5; Table S1) were used to create a single bi-ventricular geometry (i.e., a single cardiac shape). Briefly, the left ventricular apices of the three data sets were aligned, and only voxels that were common to all three reconstructions were preserved. This simple approach yielded a single hybrid bi-ventricular geometry within which the myocyte orientations could be represented by each of the three DTI-based anatomical data sets (referred to as DTI1, DTI2, and DTI3, respectively) with no registration errors or interpolation involved. As myocyte orientations in cardiac modeling studies are typically based on two values of the diffusion coefficient (longitudinal and transverse), axially symmetric anisotropy was assumed for all DTI-based myocyte orientation scenarios (i.e., based on the primary eigenvector alone). In a preliminary study, we showed that simulated arrhythmia dynamics under anisotropic and orthotropic conditions were quantitatively similar (24), supporting the appropriateness of this simplification. Histograms of the helix and transverse angles for the newly created DTI1, DTI2, and DTI3 myocyte orientation scenarios are shown in Fig. S2, in which it can be seen that variability observed in the unprocessed data was preserved.

Rule-based myocyte orientations

As an additional comparator, a rule-based (RB) assignment of myocyte orientations was performed on the ventricular geometry, based on methods described previously (25,26). Briefly, an idealized bi-ventricular geometry, wherein the left ventricle (LV) and right ventricle (RV) were modeled as

thick- and thin-walled truncated ellipsoids, respectively, was overlain on to the hybrid bi-ventricular geometry to assign myocyte orientation at each tissue voxel. A value of the helix angle, α, was assigned by $\alpha = R(1 - 2d)$, where $R = 60^\circ$ defines transmural myocyte rotation (varying from $+R$ at the endocardium to $-R$ at the epicardium), and d is the normalized transmural depth (varying from 0 at the endocardium to 1 at the epicardium). The transverse angle was assumed to be 0° (26), consistent with the general trend in DTI data (23). A comparison of the four myocyte orientation scenarios is shown in Fig. 1 *A*.

Ventricular tissue simulations

The Fenton-Karma three variable (FK3V) minimal AP model (27) was modified to reproduce the short rat ventricular AP duration (APD) of ~50 ms and its restitution, using published data from optical mapping experiments performed in our lab (Fig. 2, *A* and *B*; (28)). The model outputs a normalized "membrane potential," u, between 0 and 1, which can be scaled to a physiological range of membrane potentials using $V = V_0 + u(V_f - V_0)$, where V_0 and V_f, which correspond to the resting membrane potential and Nernst potential of the fast inward current, respectively, are set to −85 and +15 mV (27). The modified FK3V model parameter values are given in Table 1 (see (27) for a full list of model equations). Whereas, in general, we wanted to minimize non-structural determinants of model behavior, during rapid pacing (see Protocol 2—re-entry inducibility), arrhythmia induction was not possible in homogeneous simulations because of uniform conduction block (29), so it was necessary to introduce electrophysiological heterogeneity (between the LV and RV and from apex to base). Briefly, the τ_r parameter in the FK3V model was increased linearly by 20% from apex to base and was reduced by 20% in the RV compared with LV. This produced a longer APD in the LV than RV and longer APD at the base compared with apex (see Fig. S3 for regional restitution curves and AP profiles), consistent with experimental observations (30).

Propagation of APs in tissue was described using the monodomain equation as follows:

$$\frac{\partial V}{\partial t} = \nabla(\mathbf{D}\nabla V) - \frac{I_{ion}}{C_m}, \quad (1)$$

where V is the transmembrane voltage, $\mathbf{D}$ is the global conductivity ("electrical diffusion") tensor, I_{ion} is the total ionic current, and C_m is the membrane capacitance. Eq. 1 was solved using a finite difference partial

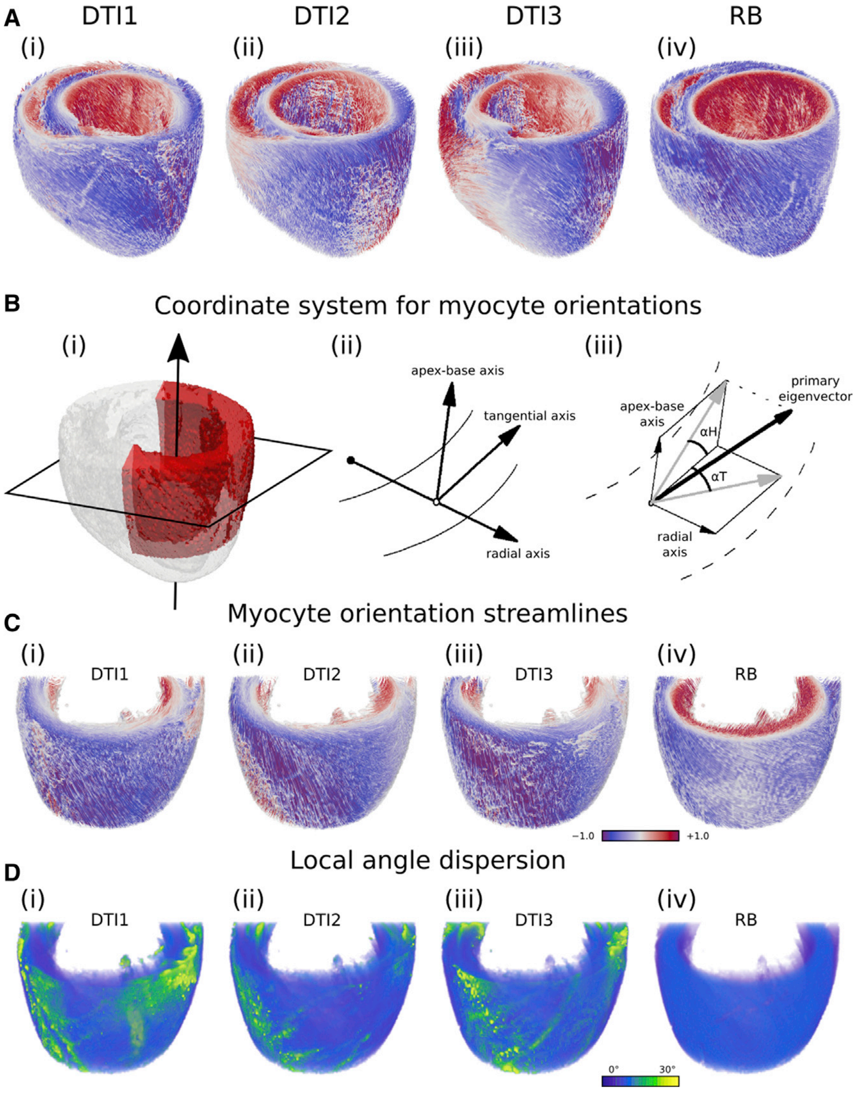

FIGURE 1 Variable myocyte orientation models. (*A*) Shown is the single hybrid bi-ventricular geometry used in this study, with variable myocyte orientations based on three DTI data sets (i–iii) and idealized RB myocyte orientations (iv). Streamlines are colored using a custom cyclic scheme according to the *z* component of the myocyte orientation, which coincides with the apico-basal (*long*) axis, such that the color red indicates that the orientation is pointing up toward the base, the color blue down toward the apex, and the color white along the short axis plane. (*B*) Coordinate system used to compute myocyte orientation angles is shown. (i) A base-apex axis is fitted to the center of the LV, normal to the transverse plane of the heart (shown as a *rectangle* in the short-axis plane). (ii) Three orthogonal reference axes are defined for each voxel, from which (iii) the helix angle and transverse angle are calculated from the primary eigenvector, and the sheetlet angle (not considered in this study) is calculated from the secondary eigenvector (5). (*C*) Myocyte orientation streamlines from the left ventricular wedge are highlighted in red in panel (*B*i) for the three DTI-based myocyte orientation scenarios (i–iii) and RB myocyte orientations (iv). (*D*) Shown are corresponding maps of the average angle between the myocyte orientation at a given tissue voxel and its neighbors (in which the maximum angle difference is 90°). Patches of green/yellow show areas of high disorganization.

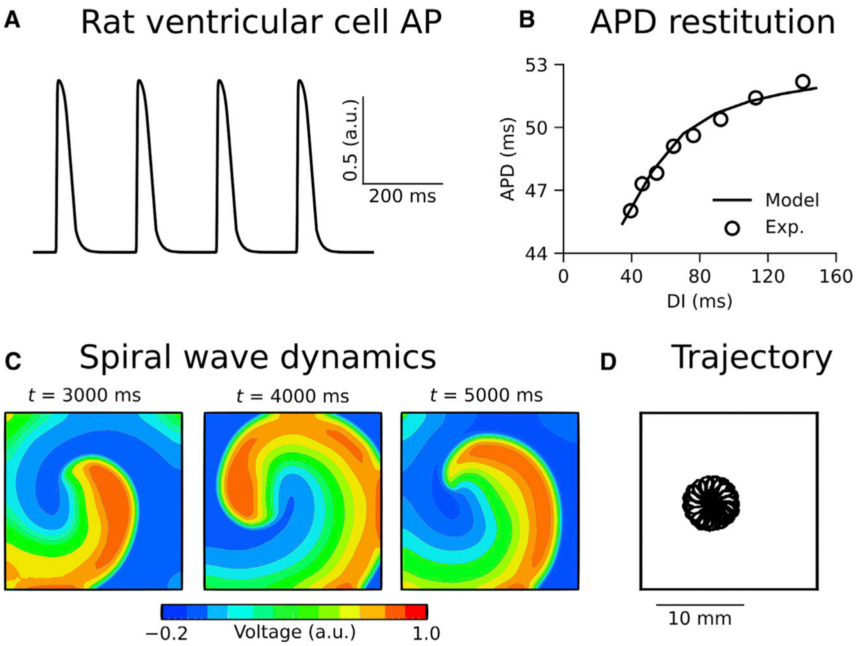

FIGURE 2 Rat ventricular electrophysiology model. (*A*) A train of rat ventricular cell action potentials (APs) using the modified FK3V model at a pacing rate of 5 Hz is shown. (*B*) Comparison of AP duration (APD) as a function of diastolic interval (DI) using a dynamic restitution protocol from model (*solid line*) and experiment (points) is shown (28). (*C*) Snapshots of spiral wave dynamics and (*D*) trajectory of the spiral wave core is shown in an isotropic two-dimensional tissue sheet.

differential equation solver based on the explicit forward Euler method with time step $\Delta t = 0.01$ ms and a Strang splitting scheme, which ensured numerical stability. The global conductivity tensor, **D**, from Eq. 1 is given by the following:

$$\mathbf{D} = D_2\mathbf{I} + (D_1 - D_2)\boldsymbol{e}_1\boldsymbol{e}_1^{\mathrm{T}}, \tag{2}$$

where D_1 and D_2 correspond to electrical diffusion in directions along and axial to the local myocyte orientation, respectively, **I** is the identity matrix, $\boldsymbol{e}_1$ is the primary eigenvector obtained from DTI (corresponding to local myocyte orientation), and the superscript T denotes the vector transpose. D_1 was set to 0.15 mm^2/ms, corresponding to a conduction velocity of 0.6 m/s along the myocyte orientation axis (31), and D_2 was scaled using the ratio D_1:D_2 = 4:1 (31) to give a conduction velocity perpendicular to the local myocyte orientation of 0.3 m/s. In an isotropic two-dimensional sheet with 200 × 200 grid points and spatial step 100 μm (to match the resolution of the 3D geometry as determined by DTI (23)), a spiral wave initiated using an S1–S2 cross-shock protocol was shown to follow a stable and stationary epicycloidal trajectory (Fig. 2 *C*). This ensured that scroll wave break in the 3D model was due to structural effects rather than membrane kinetics. The resulting reaction-diffusion model was used to probe the effects of myocyte orientations on ventricular activation and arrhythmogenesis, using three specially designed simulation protocols (next sections). Unless stated otherwise, homogeneous electrophysiology was assumed. The updated FK3V model and complete geometry files used in this study are freely accessible at (https://github.com/DGWhittaker/Rat-FK3V-files).

TABLE 1 Parameter Values for the Updated FK3V Model

Parameter	BR	Rat Ventricle Model
$\overline{g}_{fi}$	4	10*
τ_r	33.33	33.33
τ_{si}	29	42.34*
τ_0	12.5	12.5
τ_v^+	3.33	3.33
τ_{v1}^-	1250	464.15*
τ_{v2}^-	19.6	54.32*
τ_w^+	870	870
τ_w^-	41	41
u_c	0.13	0.13
u_v	0.04	0.04
u_c^{si}	0.85	0.85

A list of parameter values for the modified Fenton-Karma three variable (FK3V) model, which recapitulates the rat ventricular APD and restitution (28) compared with Beeler-Reuter (BR) (53) parameters from the original publication (27), in which differences are marked with an asterisk. For cell model equations, see (27).

Protocol 1—paced activation and repolarization

Protocol 1 was designed to assess the effects of myocyte orientation variability on ventricular activation and repolarization times during paced activation at a normal rate. For each of the four myocyte orientation scenarios (three from DTI, and one RB), the left ventricular apex was stimulated at pacing rates of 2.5, 5, and 10 Hz (corresponding to cycle lengths of 400, 200, and 100 ms, respectively), giving 12 simulations in total. Local activation time was defined as the time taken for the membrane potential to exceed a threshold value of 0.5 (corresponding to −35 mV) at each node, and repolarization time was the time at which the membrane potential returned to a threshold value of 0.1 (corresponding to −75 mV). Each of these was measured during the final excitation from a train of 10 APs.

Protocol 2—re-entry inducibility

Protocol 2 was designed to assess the effects of myocyte orientation variability on re-entry inducibility at rapid pacing rates. Conduction velocity was reduced for this protocol (by 50%) to facilitate sustenance of re-entry while also simulating gap junction remodeling that can occur under pathological conditions. Five locations on the ventricular geometry (LV and RV apex, two locations on the LV free wall, and the RV base; illustrated in Fig. S4) were stimulated across a range of rapid pacing cycle lengths (from 50 to 70 ms in 2 ms intervals, corresponding to a range of 14.28–20 Hz—consistent with VF rates in rat (32)) to attempt to induce sustained re-entrant excitations (giving a total of 220 simulations). The resulting behavior for each stimulus-location/cycle-length pair was classed as either 1) normal propagation, 2) propagation block, 3) non-sustained

arrhythmia, or 4) sustained arrhythmia (defined as lasting for the 5000 ms duration of simulations). We defined propagation block as failure to capture all stimuli. As re-entry could not be induced by rapid pacing using the rat FK3V model under homogeneous conditions because of uniform conduction block (29), it was necessary to introduce heterogeneous electrophysiology for this protocol, as described in Ventricular tissue simulations.

Protocol 3—long-term and average arrhythmia dynamics

Protocol 3 was designed to assess the effects of myocyte orientation variability on arrhythmia dynamics (as quantified by scroll wave filament analysis) averaged over multiple episodes to assess any overall differences in the ability to sustain arrhythmia between the three anatomical models and the one idealized model utilized. The phase distribution method (33–35) was used to initiate a scroll wave, which developed into re-entry at 10 different locations (two on each of the left ventricular apex, left ventricular lateral, anterior, and posterior walls, and the right ventricular wall) for each of the four myocyte orientation scenarios, giving 40 simulations in total. As the focus of this protocol was on arrhythmia dynamics rather than inducibility, conduction velocity was decreased by a factor of three (through a ninefold reduction in the diffusion coefficient) to strongly facilitate sustenance of re-entry in the limited ventricular mass.

Scroll wave filaments, which act as "organizing centers" of scroll waves, were tracked using the method of locating phase singularities (36). Briefly, a transformation into phase space was established by point-wise time-delay embedding of the transmembrane potential, wherein a phase angle about the origin (V^*,V^*) was calculated, given by the following:

$$\varphi(t) = a\tan 2(V(t) - V^*, V(t-\tau) - V^*), \tag{3}$$

where $a$tan2 is a four-quadrant inverse tangent such that $\varphi(t)$ is returned in the desired range $[-\pi,\pi]$, V^* is the activation threshold (set to 0.5 or −35 mV), and τ is the time delay, which was set to 10 ms (37). Detection of filaments was based on locating lines of wave break about which scroll waves rotate (9); at phase singularity points, which form these lines, or "filaments," the phase is undefined. Once phase singularities were located, scroll wave filaments were counted using a grassfire algorithm (38). As single filaments can be broken into multiple filaments by the intricate myocardial structure (2), in addition to the mean number of filaments, the total filament length was determined (the sum of the lengths of all individual filaments), in which we computed the mean (averaged over the re-entry lifespan) and maximum (the maximal value registered in each simulation) to quantify wave activity. These values were compared with one-way analysis of variance followed by Bonferroni post hoc tests in R. Results were deemed statistically significant if $p < 0.05$. An example of arrhythmia initiation using the phase distribution method, along with corresponding scroll wave filaments and measures of the filament dynamics, is shown in Fig. S5.

RESULTS

A comparison of the four myocyte orientation scenarios is given in Fig. 1, which shows streamlines colored according to the z component of the myocyte orientation (which coincides with the apico-basal axis). It can be seen here that whereas there was a predictable organization to the DTI-based myocyte orientations (varying from red at the endocardium to blue at the epicardium, corresponding to transmural rotation of myocyte orientations), there were also discontinuities and patches that were not captured by the idealized RB myocyte orientations. Maps of the mean angle between the myocyte orientation at a given tissue voxel and its neighbors (in which the maximal angle difference is 90°) for each of the myocyte orientation scenarios in a left ventricular wedge provides quantification of these features (Fig. 1, *B–D*). Whereas this angle was generally <10° throughout the ventricles for the RB approximation, for the DTI-based scenarios, there existed patches of myocyte disorientation with abrupt changes of >30°.

Protocol 1—paced activation and repolarization

Total activation times (i.e., the time taken after the stimulus is applied for all nodes in the tissue to become active) were consistent between the four myocyte orientation scenarios (Fig. 3, *A* and *B*), with maximal deviations of 1, 2, and 2 ms for pacing frequencies of 2.5, 5, and 10 Hz, respectively. Total repolarization times were also quantitatively similar, with a maximal divergence between myocyte orientation scenarios of 1 ms across all pacing rates. In supplementary simulations that included heterogeneous electrophysiology, the maximal divergence in activation times remained the same, whereas maximal repolarization heterogeneity increased to 4 ms (Fig. S6). Representative activations from the homogeneous case after left ventricular apical stimulation from a normal (in rat) pacing rate of 5 Hz show that patterns were qualitatively similar between the four myocyte orientation scenarios. Video S1 shows these activations more clearly, in which it can be seen that although total activation times were similar, wavefront curvature differed between myocyte orientation scenarios, giving rise to differences in local activation time.

Analysis of differences between each myocyte orientation scenario showed that mean absolute differences in activation time were quantitatively similar (Table 2). Although this value increased with the pacing rate, it remained in all cases ≤3.73 ms. The biggest divergence in activation times was between RB and DTI3, whereas the smallest divergence in activation times was between DTI2 and DTI3. Relative mean absolute differences in local activation times could also, under some circumstances, depend on pacing rate; for example, at 2.5 Hz, the difference between DTI1 and RB was bigger than the difference between DTI1 and DTI2, whereas at 10 Hz, the converse of this was true.

To see whether differences in local activation time corresponded to regions of myocyte disorientation, we visualized the myocyte orientations in the RB and DTI-based myocyte orientation scenarios, along with the corresponding absolute difference in local activation time in a wedge of the ventricles, which showed the greatest difference (Fig. 3, *C* and *D*). The RB scenario, which shows the most smooth and orderly myocyte orientations, was chosen as a "control" to facilitate comparison. It can be seen that differences in local activation time aligned well with areas of myocyte disorientation. This is particularly evident for DTI2 and DTI3, where differences in local activation time of up to 9 ms coincided with areas of irregular, discontinuous myocyte organization.

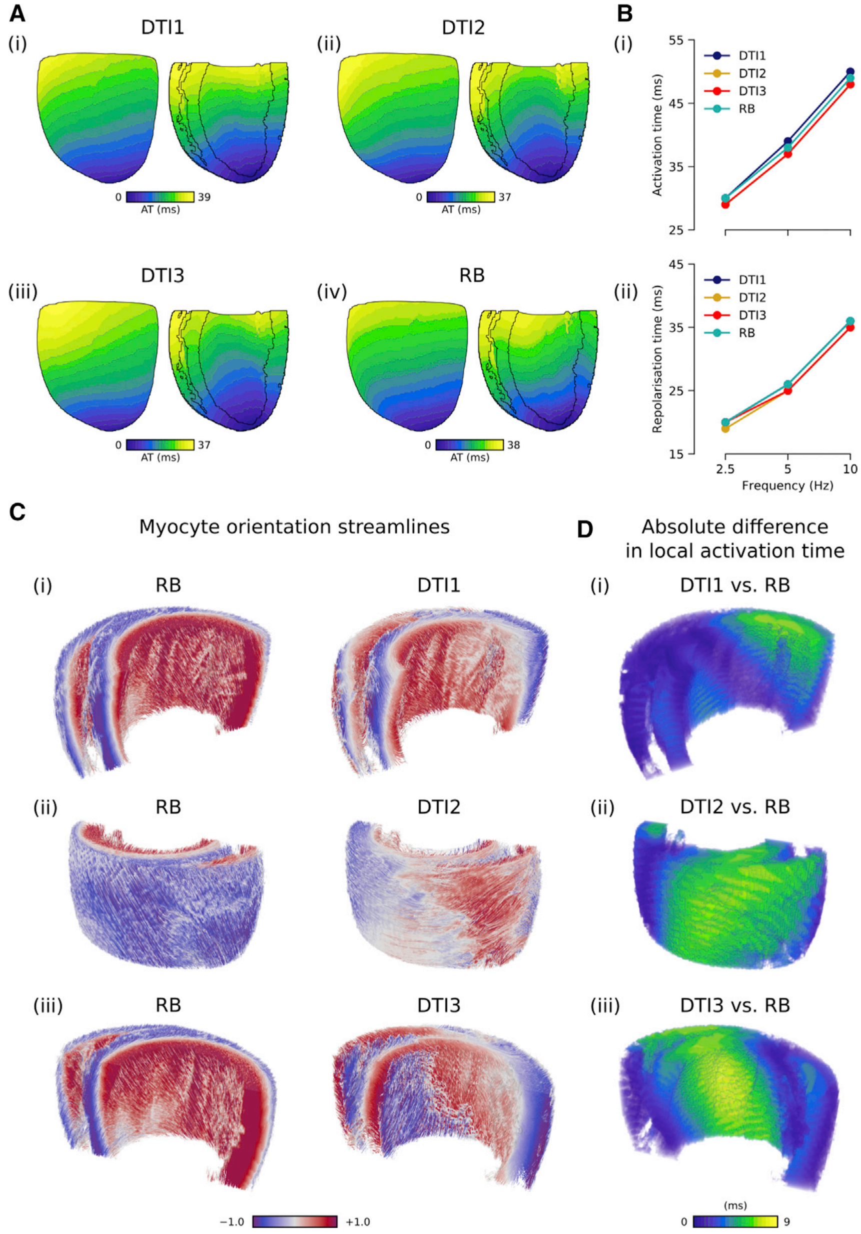

FIGURE 3 Effects of myocyte orientations on activation time. (*A*) Activation patterns at 5 Hz for (i) DTI1, (ii) DTI2, (iii) DTI3, and (iv) RB myocyte orientation scenarios are shown from a left ventricular posterior wall view and a cross-sectional view into the RV and LV cavities. (*B*) Shown is a summary of total (i) activation and (ii) repolarization times for all myocyte orientation scenarios at pacing rates of 2.5, 5, and 10 Hz (corresponding to cycle lengths of 400, 200, and 100 ms, respectively). (C) Shown are myocyte orientation streamlines in a bi-ventricular wedge for RB and (i) DTI1, (ii) DTI2, and (iii) DTI3 myocyte orientation scenarios. (*D*) Shown are corresponding absolute differences in local activation time, taken from the whole bi-ventricular geometry simulations at 5 Hz.

Protocol 2—re-entry inducibility

Arrhythmic conduction patterns could be induced at least once within each of the myocyte orientation scenarios, although the long-term behavior and stimulus-location/cycle-length pairs over which they could be induced varied between scenarios. An example of a condition in which re-entry was inducible for one myocyte orientation scenario (DTI2) but not others is shown in Fig. 4 *A* (based on rapid pacing at a cycle length of 60 ms at the RV apex). Under these conditions, small differences in cellular organization translated to whether or not re-entrant excitations could be induced for this particular protocol. Video S2 and AP time series recorded from the LV apex show that DTI2 allowed re-entrant activity to develop in the ventricles by capturing the third stimulus in which others did not, which led to non-uniform conduction block and subsequent re-entry. Summarizing the stimulus-location/cycle-length pairs over which one of the four classifications of dynamics emerged, pertaining to 1) normal propagation, 2) propagation block, 3) non-sustained arrhythmia, or 4) sustained arrhythmia, demonstrates the differences between the scenarios (Fig. 4 *C*). Although differences between myocyte orientation scenarios were apparent, the patterns showed some similarity and consistency within each location investigated. For example, there was agreement across all scenarios that the RV base was an area of high arrhythmia inducibility,

TABLE 2 A Summary of Absolute Differences in Activation Time for Protocol 1

	DTI1	DTI2	DTI3
DTI2	1.17/1.61/2.36	–	–
DTI3	1.47/2.11/3.15	0.55/0.85/1.36	–
RB	1.30/1.62/2.19	1.89/2.49/3.46	1.98/2.68/3.73

A summary of mean absolute differences in local activation time between myocyte orientation scenarios for different pacing rates. Data are presented in order of increasing pacing rate (i.e., values of the mean absolute difference at 2.5/5/10 Hz).

whereas the LV free wall location 2 was an area of low/no inducibility. The cycle length at which the transition from normal propagation to propagation block occurred was also highly similar in most cases, with a maximal divergence of 4 ms (for one instance only at the RV apex).

Table S2 summarizes whether or not an arrhythmia was inducible (i.e., category (3) or (4)). The RB scenario was the only one for which an arrhythmia could be induced at all locations. However, considering the total number of arrhythmias inducible across all five locations, the RB scenario was the second least prone to arrhythmia induction (after DTI1). Table S2 highlights a large effect of natural variability in myocyte orientations; there were three stimulus-location/cycle-length pairs for which an arrhythmia could be induced at the LV apex for DTI2 compared with none for DTI1. This protocol demonstrates the importance of myocyte orientations on specific arrhythmia dynamics.

Protocol 3—long-term and average arrhythmia dynamics

Finally, we sought to determine to what extent myocyte orientations influence overall arrhythmia dynamics in the three anatomical models, as quantified by scroll wave filament analysis over multiple simulations. A representative arrhythmia simulation, in which a re-entrant scroll wave was initiated at the LV apex, is shown in Fig. 5 *A*. It can be seen that, in this case, myocyte orientation differences between simulations produced divergent behavior within a short time frame (200–500 ms) and could account for whether the initiated scroll wave self-terminated, remained VT-like (single scroll wave filament), or degenerated into VF-like activity (multiple scroll wave filaments). This is further highlighted in Video S3, in which the dynamic evolution of scroll waves and their organizing centers (filaments) are shown over a 1000 ms time frame. However, when averaging over 10 simulations (i.e., over the 10 different re-entry initiation sites), measures of the arrhythmia dynamics (specifically, the mean number of filaments, mean total filament length, and maximal total filament length) were quantitatively similar; for example, the mean total filament length was in the range of 1.0–1.5 mm for all four microstructure scenarios. Fig. 5 *C* shows a summary of these measures; no statistically significant differences between values obtained from the four different myocyte orientation scenarios were observed (one-way analysis of variance *p*-values are shown in Table S3).

DISCUSSION

Main findings

In this study, the role of variability in cardiac myocyte orientations in ventricular activation and arrhythmogenesis was investigated using reaction-diffusion models of cardiac electrophysiology with modified FK3V membrane kinetics and a novel image-based myocardial geometry from high-resolution DTI (23), in which the myocyte orientations could be changed to one of four scenarios.

We initially hypothesized that intersubject variability in myocyte orientations plays an important role in determining ventricular arrhythmogenic wave dynamics. Our main findings in this regard were that myocyte orientations can have a large influence on local and specific dynamics: 1) differences in local activation time observed between the four scenarios aligned with areas of myocyte disorientation; and 2) myocyte orientations alone led to crucial differences in the inducibility and persistence of arrhythmias at different locations across the ventricles and were able to account for important differences in arrhythmia dynamics, such as whether simulated scroll waves self-terminated, remained VT-like, or degenerated into VF-like activity. However, in the context of global/average behavior, our findings indicate that myocyte orientations had only a small impact; total activation times and overall susceptibility to arrhythmia were similar for the four different scenarios.

Impact of myocyte orientation variability on normal propagation

Our results from Protocol 1 suggest that variation in myocyte orientations had a minimal influence on excitation-propagation at normal pacing rates, in agreement with previous human atrial simulations (39). The maximal mean absolute difference in local activation times between myocyte orientation scenarios was 2.68 ms at a pacing rate of 5 Hz but could be as low as 0.85 ms (Table 2). However, an important rate dependence was observed wherein differences were accentuated by pacing rate. This suggests that the faster the electrical activity to be investigated, the more important sample-specific myocyte orientations become. This was supported by Protocol 2, in which significant, unpredictable discrepancies in arrhythmia inducibility between the different myocyte orientation scenarios at rapid pacing rates occurred because of enhanced repolarization heterogeneity. This has implications for arrhythmogenesis in cardiac pathologies, which are associated with the

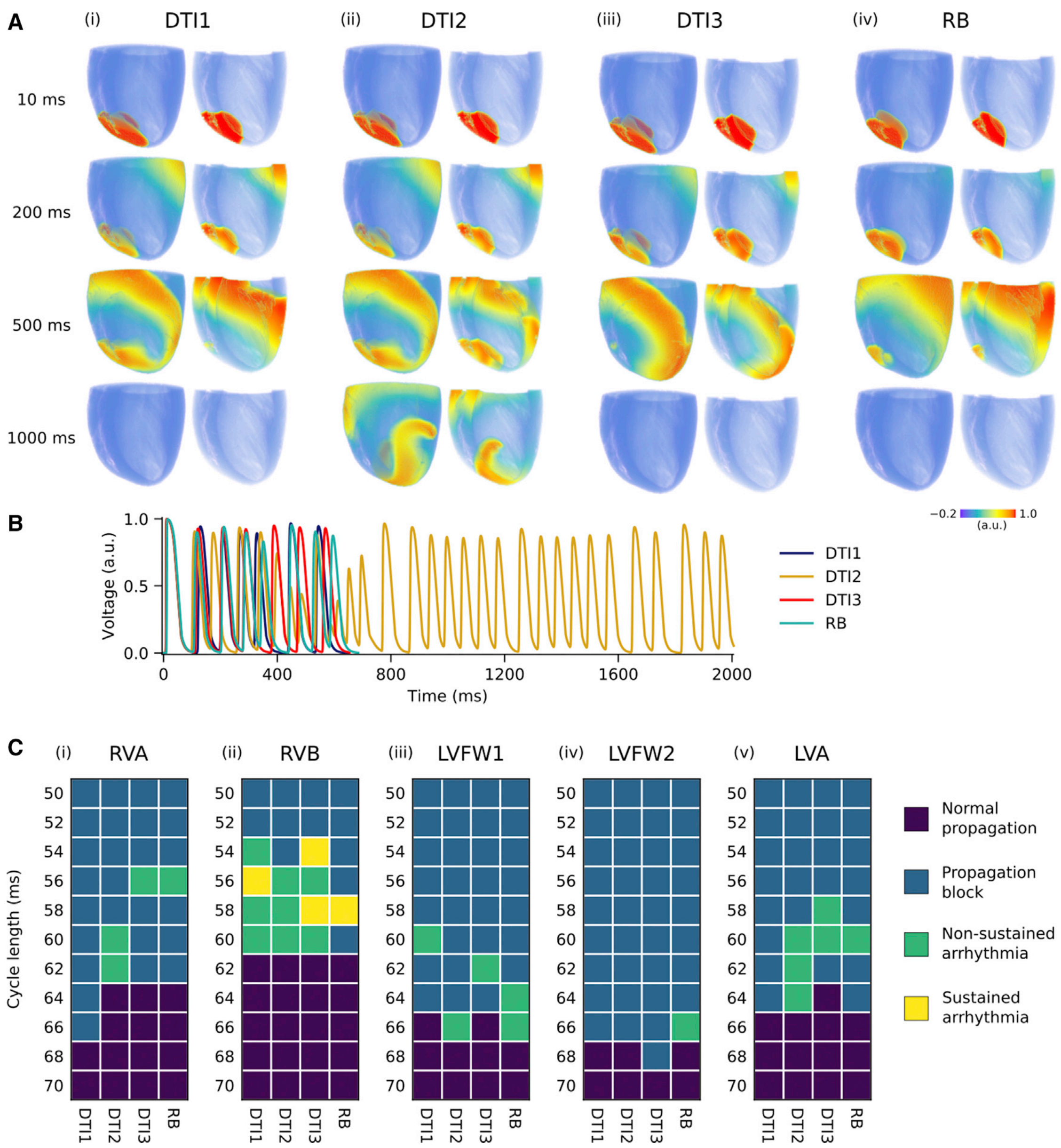

FIGURE 4 Effects of myocyte orientations on arrhythmia inducibility at rapid pacing rates. (*A*) Shown are representative snapshots of membrane potential for the (i) DTI1, (ii) DTI2, (iii) DTI3, and (iv) RB myocyte orientation scenarios at a pacing cycle length of 60 ms at the RV apex, shown from a left ventricular posterior wall view, and a cross-sectional view into the RV and LV cavities. (*B*) Corresponding AP time series recorded over 2000 ms from the LV apex. (*C*) Shown are vulnerability grids for arrhythmia inducibility after rapid pacing across a range of cycle lengths at the (i) right ventricular apex (RVA), (ii) right ventricular base (RVB), (iii) left ventricular free wall location 1 (LVFW1), (iv) left ventricular free wall location 2 (LVFW2), and left ventricular apex (LVA).

disorganization of myocyte orientations (28), suggesting that altered cellular organization may become a critical substrate for re-entry associated with fast pacing rates. Furthermore, conditions that promote discontinuities in myocyte orientations may result in regions of higher vulnerability to re-entry induction, which requires subject-specific myocyte orientations to investigate on a patient-specific basis.

Interestingly, differences between different DTI-based myocyte orientation scenarios could be larger than the differences between DTI-based and idealized RB myocyte orientations. For instance, the mean absolute difference in local activation times between DTI1 and DTI3 was larger than the difference between DTI1 and RB. This calls attention to the large degree of natural variability in myocyte orientations (40,41), which can be patchy and discontinuous or relatively smooth and organized like the idealized scenario. Furthermore, this finding highlights the ability of idealized approaches to capture the dynamics of ventricular activation under conditions of relatively organized myocyte orientations.

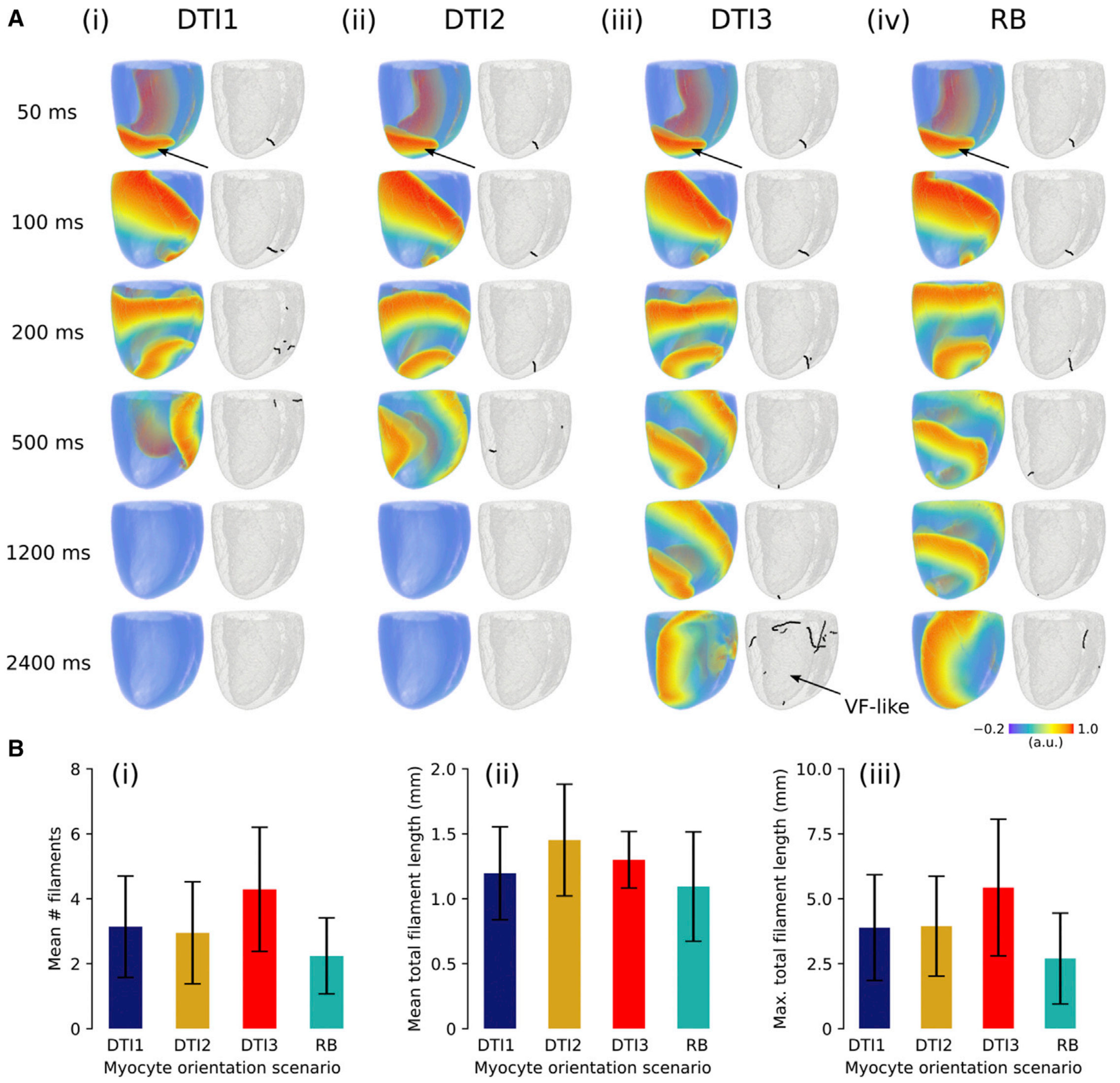

FIGURE 5 Effects of myocyte orientations on overall arrhythmia dynamics. (*A*) Shown is a left ventricular anterior wall view of the evolution of a scroll wave initiated on the left ventricular apex (marked with an *arrow*) for (i) DTI1, (ii) DTI2, (iii) DTI3, and (iv) RB myocyte orientation scenarios, with corresponding scroll wave filaments shown as black lines on a semi-transparent contour of the geometry. The arrow for DTI3 at $t = 2400$ ms highlights VF-like activity. (*B*) Shown are summary data of (i) mean number of filaments and (ii) mean and (iii) maximal total filament length across different myocyte orientation scenario re-entry simulations ($n = 10$ for each scenario). Results are expressed as mean $\pm$ SD.

Impact of myocyte orientation variability on arrhythmogenesis

Protocol 2 revealed that variation in myocyte orientations led to critical differences in arrhythmia inducibility at different locations across the ventricles. One reason for this may be that pacing near discontinuities in myocyte organization can cause conduction block (7). Myocyte orientation disorganization is clearly only one factor that determines arrhythmogenicity, however. Another determinant of arrhythmogenic wave dynamics is the interaction of cellular organization with heterogeneous electrophysiology, in which configurations at critical locations may be favorable to the development of re-entry pathways. For example, it was shown previously using a high-resolution ($\sim$70 μm) micro-CT-based computational model of the canine left atrium and pulmonary veins that disorganized myocyte orientations combined with electrophysiological heterogeneity at this junction promoted conduction block and subsequent re-entry (7). As we investigated behavior only at a limited number of stimulus-location/cycle-length pairs, we uncovered only a small fraction of the effects of myocyte orientation variability, which was nonetheless significant.

When taken as a whole, the vulnerability grids in Fig. 4 showed some consistency between the myocyte orientation scenarios regarding whether each location was one of high

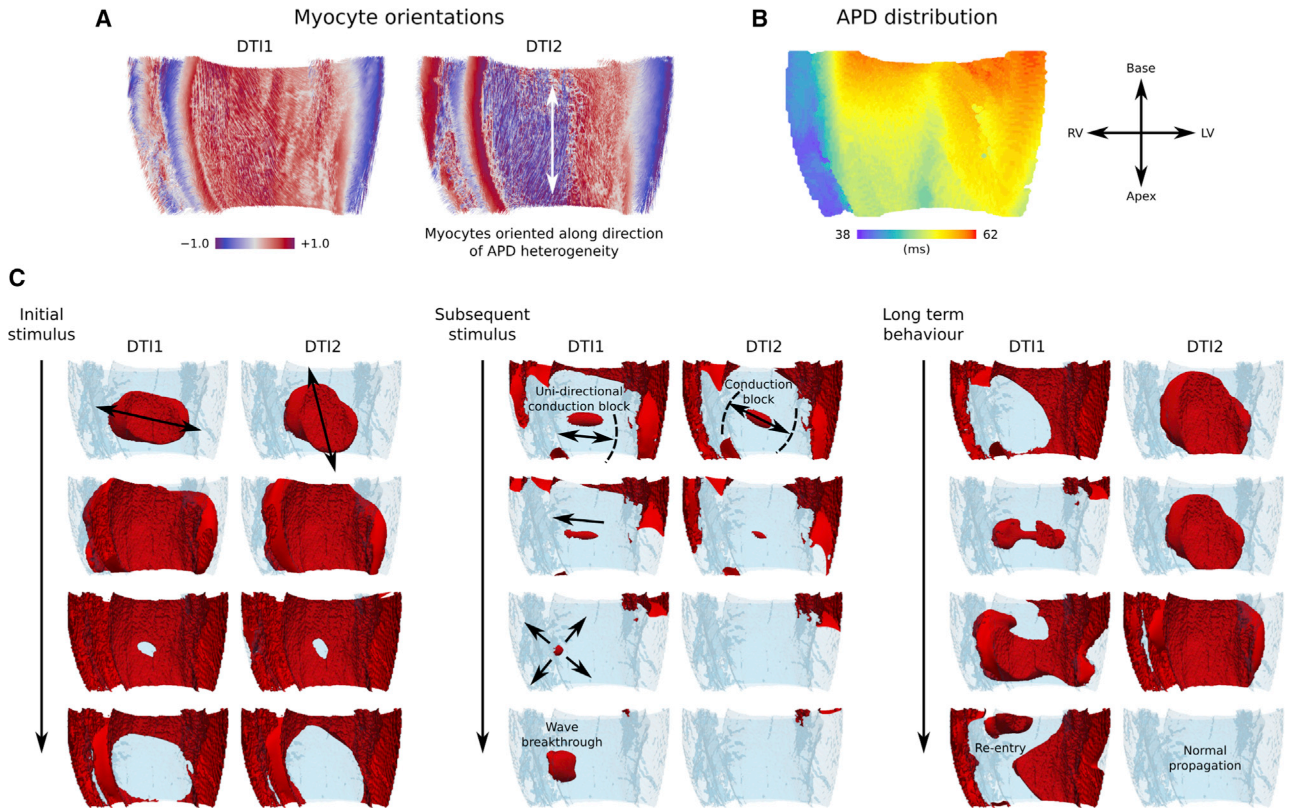

FIGURE 6 A mechanism by which differences in myocyte organization directly influence arrhythmia inducibility. (*A*) Myocyte orientation streamlines for DTI1 and DTI2 in a bi-ventricular wedge are shown. (*B*) AP duration (APD) distribution after left ventricular free wall stimulation is shown. (*C*) Shown are snapshots of the evolution of wave dynamics after repeated stimulation of the left ventricular free wall (LVFW1) in Protocol 2, in which excited tissue is shown in red on a semi-transparent contour of the geometry. Differences in myocyte orientations influence the direction in which the wave propagates and repolarizes after the initial stimulus. After the subsequent stimulus, the wave propagates preferentially toward the basal region of longer APD for DTI2 and thus is blocked, whereas for DTI1, the wave propagates slightly more toward the RV region of short APD, and thus, a narrow wave breakthrough occurs. This leads to a significant asymmetry in the activation-recovery cycle and eventual development of re-entry for DTI1 but not DTI2.

or low arrhythmia inducibility and also the pacing rate at which the transition to propagation block occurred. However, important differences in arrhythmia inducibility arose under certain conditions; for example, at a pacing rate of 60 ms at the LV apex, an arrhythmia could be induced for all scenarios except DTI1, whereas at a pacing rate of 60 ms at the LV free wall location 1, an arrhythmia could be induced for the DTI1 scenario only. Looking at this specific example in more detail, by comparing DTI1 and DTI2, revealed a critical combination of myocyte organization and electrophysiological heterogeneity that allowed the development of significant asymmetry in the activation-recovery cycle and subsequent development of re-entry in DTI1 but not DTI2 (Fig. 6; Video S4). For comparison, under isotropic conditions, no arrhythmias could be induced at the RV base, the area of highest arrhythmia inducibility, which confirms that myocyte orientations were an important factor underlying arrhythmogenesis in the model (Fig. S7).

Protocol 3 highlighted the fact that in the absence of differences in electrophysiology, myocyte orientations alone could account for a large variation in arrhythmia dynamics, critically determining whether scroll waves self-terminated, remained VT-like, or degenerated into fibrillatory activity from the same initial conditions. These findings suggest that in structurally normal hearts, in which cellular organization is intact, it is difficult to predict the lifespan or location of scroll waves from generic re-entry simulations (in contrast to, for example, postinfarction hearts, in which re-entrant circuits tend to anchor to scars (20)). This may have important implications for safety-critical applications, such as the prediction of re-entry pathways or optimal ablation sites in patients in which the re-entrant substrate is due to excitable microstructural discontinuities (rather than unexcitable scar tissue) or is purely electrophysiological (e.g., under genetic mutation or pharmacological modulation (11,14)).

An assessment of arrhythmia dynamics arising from 10 different re-entry simulations (i.e., averaged over the 10 different re-entry initiation sites) revealed that, for these three DTI-based data sets and the idealized RB case, scroll wave filament dynamics were quantitatively similar under conditions of different myocyte orientations, and no statistically significant differences in measures of the filament dynamics between the different scenarios were found. This is

consistent with a preliminary work, in which we also showed that measures of arrhythmia dynamics were not significantly different between similar-sized hearts, each with sample-specific myocyte orientations (24). Thus, in the limited case of investigating three hearts and an idealized case only, no clear intersubject variability in overall sustainability of arrhythmia was observed; such results, however, cannot be extrapolated to general statements about population variability nor to potential arrhythmia variability in diseased conditions, in which intersubject variation of myocyte orientations may be larger than in control conditions. For example, remodeled hearts in diseases such as heart failure may show greater heterogeneity of myocyte disorganization and thus larger differences in global/average behavior.

Nonetheless, our data do suggest that simulations at multiple locations should be performed for a thorough assessment of cardiac arrhythmogenicity and further support the value and validity of using idealized, RB approximations for general mechanistic investigation. To probe overall arrhythmogenicity associated with non-structural alterations in the heart (e.g., drug- or genetic mutation-induced effects), a similar-sized reference geometry with non-specific myocyte orientations can provide useful predictions. This is already a common approach in cardiac modeling (10,12,34,37) and is often a necessity because of absent or low-resolution patient data (21). However, where specificity is desired (e.g., when predicting location-dependent arrhythmia inducibility or re-entry pathways on a patient-specific basis), the importance of subject-specific myocyte orientations emerges.

Clinical relevance

Basic research remains fundamental to gaining mechanistic insights into the role of cardiac architecture on the complex spatiotemporal activity of arrhythmias (42–44). However, models of cardiac activity are now also moving to applications in the clinic as the synergy between imaging and computational models continues to enable novel investigations in cardiovascular research (21). Our results indicate that sample-specific myocyte orientations may be necessary to quantitatively predict arrhythmia inducibility on a subject-specific basis. This has implications for clinical-modeling pipelines; attaining the highest possible resolution microstructural information should be a priority for the purposes of arrhythmia risk quantification. Whereas several cardiac imaging modalities are available, such as echocardiography, MRI, and computed tomography (CT), each with associated advantages and disadvantages, none yet possess the capability to obtain high-resolution myocyte orientations in vivo at the microscopic scale (21), although advances have been made in in vivo human data acquisition using DTI (45,46). Continuing advances in obtaining myocyte orientations in vivo will facilitate characterization of its role and proarrhythmic potential in cardiac pathologies. Another relevant aspect of our study to consider here is that activation patterns were quantitatively similar for all myocyte orientation scenarios, suggesting that it is very challenging or effectively impossible to infer myocyte orientations from electrocardiogram or electrode mapping. This further supports the use of DTI in the clinic as a means of characterizing tissue microstructure.

As cardiac modeling is moving increasingly toward patient-specific evaluations (47), it is likely that advances in preclinical and clinical imaging technologies providing high-resolution anatomy and myocyte orientations and integration with computational models of cardiac electrophysiology, fluid dynamics, and mechanics will drive the next generation of predictive models (21). In particular, integrating personalized myocardial structure, the importance of which is demonstrated in our study, with patient-specific electrophysiology will be a crucial step toward the future translation of computational models of the heart to the clinic.

Limitations

First, because the focus of the study was cardiac structure, biophysically detailed models of rat ventricular electrophysiology (e.g., (48,49)) were not used. Instead, we opted to use a modified FK3V model, which enabled simulation of the rat APD and its restitution in a more computationally efficient manner. It is well known that electrotonic interactions in tissue influence the AP (31), the precise morphology of which in rat ventricles was likely not captured by the minimal model. As we aimed to eliminate all unnecessary non-structural determinants of arrhythmia dynamics, however, this simplification was deemed appropriate.

Second, in the absence of available multiple high-resolution data sets from humans or larger mammals for the study of variability, we used rat ventricular data. Although the helical arrangement of myocytes shows similarity between all mammalian hearts (5), there are nonetheless differences in myocyte organization and gross morphology between mammals (50), including rats and humans.

Our method of generating the single bi-ventricular structure resulted in a geometry that was not faithful to any of the individual DTI experiments on which the three DTI-based myocyte orientation scenarios were based and may have led to the loss of fine anatomical features (vasculature and endocardial structures). Nonetheless, the role of such fine structures in overall tachy- and fibrillatory-arrhythmia dynamics has been suggested to be small (51), so this is unlikely to affect fundamental conclusions drawn in our study. Similarly, this method meant that the three most similar hearts were selected; it is entirely possible that our results indicating that myocyte orientation has only a small effect on average dynamics would no longer hold if a larger set of more differentiated geometries were used, and these

statements should be interpreted in this context. Conversely, however, this limitation further supports our results regarding specific differences as these critical differences were observed even in three similar structural models and would likely be accentuated in models with greater differences. An alternative approach for our study would have been to use a standardized ventricular coordinate system (e.g., (52)) to map the myocyte orientations onto a reference geometry, although this approach does involve some small registration errors.

For the purposes of studying the effects of myocyte orientations on arrhythmic/fibrillatory behavior in the model, the diffusion coefficient, D, was reduced to favor the sustenance of such activity. It should be noted that this is equivalent to "scaling" the heart by increasing the effective tissue size. Rescaling in 3D geometries is not a simple matter as the wall thickness and heart size are both affected (4), and thus, use of different values of D in the different protocols, which were designed to address different hypotheses, somewhat limits comparison of results between protocols. Nonetheless, this does not change the conclusions drawn from individual protocols.

Finally, the method used to assign RB myocyte orientations was based on overlaying an idealized bi-ventricle model onto the real geometry. The conventional distance-map approach (18) proved to be overly complicated and awkward because of fine anatomical features, discontinuities, and nontrivial delineation of endocardial surfaces due to regions of contact between the LV and RV in rat (23). Our approach, although simplistic, reproduced the important features of RB myocyte orientations such as the smooth transmural rotation in helix angle and gave quantitatively similar activation patterns as the realistic myocyte orientations.

CONCLUSIONS

Through investigating the vulnerability to the induction of arrhythmia and its long-term behavior, we have demonstrated that myocyte organization can have a large influence on specific dynamics—to the extent to which this alone can determine whether arrhythmic conduction patterns can be induced, and for how long they persist, at specific locations. However, our results also demonstrated that overall arrhythmia dynamics—averaged across multiple initiation sites and protocols—were quantitatively similar, for the limited number of cases we studied, when using "realistic" myocyte orientations (measured using DTI) or RB assignment of myocyte orientations. We therefore highlight both the value of mechanistic studies, which implement idealized, RB approximations, and the importance of considering realistic myocyte orientations for simulations in which specificity is desired.

SUPPORTING MATERIAL

Supporting Material can be found online at https://doi.org/10.1016/j.bpj.2019.09.041.

AUTHOR CONTRIBUTIONS

D.G.W. conceived the study. D.G.W., A.P.B., and M.A.C. designed the study. D.G.W. performed numerical experiments. D.G.W., A.P.B., and M.A.C. developed computational tools. I.T. and J.E.S. contributed imaging data. D.G.W. analyzed the data. All authors contributed to the writing of the manuscript.

ACKNOWLEDGMENTS

We are grateful to Harley Stevenson-Cocks for comments on an earlier version of this manuscript. All simulations were performed on ARC3 High Performance Computing facilities at the University of Leeds, UK.

This work was funded by a Wellcome Trust Institutional Strategic Support Fund Fellowship (204825/Z/16/Z – D.G.W.) and a British Heart Foundation (BHF) project grant (PG/16/74/32374 – A.P.B.). Heart preparation and MRI scanning was funded by the Engineering and Physical Sciences Research Council (EP/J013250/1 – J.E.S.), the BHF (PG/13/33/30210 and RG/13/8/30266 – J.E.S.), and the BHF Centre for Research Excellence (RE/13/1/30181 – J.E.S.).

REFERENCES

1. Jalife, J. 2000. Ventricular fibrillation: mechanisms of initiation and maintenance. *Annu. Rev. Physiol.* 62:25–50.
2. Benson, A. P., M. E. Ries, and A. V. Holden. 2007. Effects of geometry and architecture on re-entrant scroll wave dynamics in human virtual ventricular tissues. *In* Functional Imaging and Modeling of the Heart. Springer, pp. 200–209.
3. Nanthakumar, K., J. Jalife, …, R. Dhopeshwarkar. 2007. Optical mapping of Langendorff-perfused human hearts: establishing a model for the study of ventricular fibrillation in humans. *Am. J. Physiol. Heart Circ. Physiol.* 293:H875–H880.
4. Park, S. A., and R. A. Gray. 2015. Optical mapping of ventricular fibrillation dynamics. *In* Membrane Potential Imaging in the Nervous System and Heart. M. Canepari, D. Zecevic, and O. Bernus, eds. Springer International Publishing, pp. 313–342.
5. Benson, A. P., O. Bernus, …, R. D. Walton. 2011. Construction and validation of anisotropic and orthotropic ventricular geometries for quantitative predictive cardiac electrophysiology. *Interface Focus.* 1:101–116.
6. Aslanidi, O. V., T. Nikolaidou, …, H. Zhang. 2013. Application of micro-computed tomography with iodine staining to cardiac imaging, segmentation, and computational model development. *IEEE Trans. Med. Imaging.* 32:8–17.
7. Colman, M. A., M. Varela, …, O. V. Aslanidi. 2014. Evolution and pharmacological modulation of the arrhythmogenic wave dynamics in canine pulmonary vein model. *Europace.* 16:416–423.
8. Clayton, R. H., O. Bernus, …, H. Zhang. 2011. Models of cardiac tissue electrophysiology: progress, challenges and open questions. *Prog. Biophys. Mol. Biol.* 104:22–48.
9. Clayton, R. H., E. A. Zhuchkova, and A. V. Panfilov. 2006. Phase singularities and filaments: simplifying complexity in computational models of ventricular fibrillation. *Prog. Biophys. Mol. Biol.* 90:378–398.
10. Adeniran, I., D. G. Whittaker, …, H. Zhang. 2017. In silico investigation of a KCNQ1 mutation associated with short QT syndrome. *Sci. Rep.* 7:8469.
11. Colman, M. A., E. A. Perez Alday, …, A. P. Benson. 2017. Trigger vs. substrate: multi-dimensional modulation of QT-prolongation associated arrhythmic dynamics by a hERG channel activator. *Front. Physiol.* 8:757.

12. Moreno, J. D., Z. I. Zhu, …, C. E. Clancy. 2011. A computational model to predict the effects of class I anti-arrhythmic drugs on ventricular rhythms. *Sci. Transl. Med.* 3:98ra83.

13. Ponnaluri, A. V., L. E. Perotti, …, A. Garfinkel. 2016. Electrophysiology of heart failure using a rabbit model: from the failing myocyte to ventricular fibrillation. *PLoS Comput. Biol.* 12:e1004968.

14. Whittaker, D. G., H. Ni, …, H. Zhang. 2017. Computational analysis of the mode of action of disopyramide and quinidine on hERG-linked short QT syndrome in human ventricles. *Front. Physiol.* 8:759.

15. Noble, D. 1962. A modification of the Hodgkin–Huxley equations applicable to Purkinje fibre action and pace-maker potentials. *J. Physiol.* 160:317–352.

16. Britton, O. J., A. Bueno-Orovio, …, B. Rodriguez. 2013. Experimentally calibrated population of models predicts and explains intersubject variability in cardiac cellular electrophysiology. *Proc. Natl. Acad. Sci. USA.* 110:E2098–E2105.

17. Ni, H., S. Morotti, and E. Grandi. 2018. A heart for diversity: simulating variability in cardiac arrhythmia research. *Front. Physiol.* 9:958.

18. Bayer, J. D., R. C. Blake, …, N. A. Trayanova. 2012. A novel rule-based algorithm for assigning myocardial fiber orientation to computational heart models. *Ann. Biomed. Eng.* 40:2243–2254.

19. Krueger, M. W., V. Schmidt, …, O. Dössel. 2011. Modeling atrial fiber orientation in patient-specific geometries: a semi-automatic rule-based approach. *In* Functional Imaging and Modeling of the Heart. Springer, pp. 223–232.

20. Arevalo, H. J., F. Vadakkumpadan, …, N. A. Trayanova. 2016. Arrhythmia risk stratification of patients after myocardial infarction using personalized heart models. *Nat. Commun.* 7:11437.

21. Lamata, P., R. Casero, …, V. Grau. 2014. Images as drivers of progress in cardiac computational modelling. *Prog. Biophys. Mol. Biol.* 115:198–212.

22. Le Bihan, D., J. F. Mangin, …, H. Chabriat. 2001. Diffusion tensor imaging: concepts and applications. *J. Magn. Reson. Imaging.* 13:534–546.

23. Teh, I., D. McClymont, …, J. E. Schneider. 2016. Resolving fine cardiac structures in rats with high-resolution diffusion tensor imaging. *Sci. Rep.* 6:30573.

24. Whittaker, D. G., A. P. Benson, …, M. A. Colman. 2018. Role of cardiac microstructure variability on ventricular arrhythmogenesis. *In* 2018 Computing in Cardiology Conference (CinC). IEEE.

25. Streeter, D. D., Jr., H. M. Spotnitz, …, E. H. Sonnenblick. 1969. Fiber orientation in the canine left ventricle during diastole and systole. *Circ. Res.* 24:339–347.

26. Perez Alday, E. A., D. G. Whittaker, …, M. A. Colman. 2019. Effects of heart rate and ventricular wall thickness on non-invasive mapping: an in silico study. *Front. Physiol.* 10:308.

27. Fenton, F., and A. Karma. 1998. Vortex dynamics in three-dimensional continuous myocardium with fiber rotation: filament instability and fibrillation. *Chaos.* 8:20–47.

28. Benoist, D., R. Stones, …, E. White. 2014. Systems approach to the study of stretch and arrhythmias in right ventricular failure induced in rats by monocrotaline. *Prog. Biophys. Mol. Biol.* 115:162–172.

29. Bishop, M. J., E. J. Vigmond, and G. Plank. 2013. The functional role of electrophysiological heterogeneity in the rabbit ventricle during rapid pacing and arrhythmias. *Am. J. Physiol. Heart Circ. Physiol.* 304:H1240–H1252.

30. Watanabe, T., L. M. Delbridge, …, T. F. McDonald. 1983. Heterogeneity of the action potential in isolated rat ventricular myocytes and tissue. *Circ. Res.* 52:280–290.

31. Walton, R. D., A. P. Benson, …, O. Bernus. 2013. Electrophysiological and structural determinants of electrotonic modulation of repolarization by the activation sequence. *Front. Physiol.* 4:281.

32. Handa, B. S., C. H. Roney, …, F. S. Ng. 2018. Analytical approaches for myocardial fibrillation signals. *Comput. Biol. Med.* 102:315–326.

33. Biktashev, V. N., and A. V. Holden. 1998. Reentrant waves and their elimination in a model of mammalian ventricular tissue. *Chaos.* 8:48–56.

34. Colman, M. A., H. Ni, …, H. Zhang. 2017. In silico assessment of genetic variation in KCNA5 reveals multiple mechanisms of human atrial arrhythmogenesis. *PLoS Comput. Biol.* 13:e1005587.

35. Whittaker, D. G., H. Ni, …, H. Zhang. 2017. Atrial arrhythmogenicity of KCNJ2 mutations in short QT syndrome: insights from virtual human atria. *PLoS Comput. Biol.* 13:e1005593.

36. Bray, M. A., and J. P. Wikswo. 2002. Use of topological charge to determine filament location and dynamics in a numerical model of scroll wave activity. *IEEE Trans. Biomed. Eng.* 49:1086–1093.

37. Whittaker, D. G., M. A. Colman, …, H. Zhang. 2018. Human atrial arrhythmogenesis and sinus bradycardia in *KCNQ1*-linked short QT syndrome: insights from computational modelling. *Front. Physiol.* 9:1402.

38. Clayton, R. H. 2009. Influence of cardiac tissue anisotropy on reentrant activation in computational models of ventricular fibrillation. *Phys. Nonlinear Phenom.* 238:951–961.

39. Fastl, T. E., C. Tobon-Gomez, …, S. A. Niederer. 2018. Personalized computational modeling of left atrial geometry and transmural myofiber architecture. *Med. Image Anal.* 47:180–190.

40. Gilbert, S. H., A. P. Benson, …, A. V. Holden. 2007. Visualisation of dog myocardial structure from diffusion tensor magnetic resonance imaging: the paradox of uniformity and variability. *In* Functional Imaging and Modeling of the Heart. F. B. Sachse and G. Seemann, eds. Springer, pp. 403–412.

41. Lombaert, H., J.-M. Peyrat, …, P. Croisille. 2012. Variability of the human cardiac laminar structure. *In* Statistical Atlases and Computational Models of the Heart. Imaging and Modelling Challenges. O. Camara, E. Konukoglu, M. Pop, K. Rhode, M. Sermesant, and A. Young, eds. Springer, pp. 160–167.

42. Pravdin, S. F., V. I. Berdyshev, …, V. S. Markhasin. 2013. Mathematical model of the anatomy and fibre orientation field of the left ventricle of the heart. *Biomed. Eng. Online.* 12:54.

43. Pathmanathan, P., and R. A. Gray. 2015. Filament dynamics during simulated ventricular fibrillation in a high-resolution rabbit heart. *BioMed Res. Int.* 2015:720575.

44. Konovalov, P. V., S. F. Pravdin, …, A. V. Panfilov. 2016. Scroll wave dynamics in a model of the heterogeneous heart. *JETP Lett.* 104:130–134.

45. Nielles-Vallespin, S., C. Mekkaoui, …, D. Firmin. 2013. In vivo diffusion tensor MRI of the human heart: reproducibility of breath-hold and navigator-based approaches. *Magn. Reson. Med.* 70:454–465.

46. Nielles-Vallespin, S., Z. Khalique, …, D. J. Pennell. 2017. Assessment of myocardial microstructural dynamics by in vivo diffusion tensor cardiac magnetic resonance. *J. Am. Coll. Cardiol.* 69:661–676.

47. Gray, R. A., and P. Pathmanathan. 2018. Patient-specific cardiovascular computational modeling: diversity of personalization and challenges. *J. Cardiovasc. Transl. Res.* 11:80–88.

48. Pandit, S. V., R. B. Clark, …, S. S. Demir. 2001. A mathematical model of action potential heterogeneity in adult rat left ventricular myocytes. *Biophys. J.* 81:3029–3051.

49. Gattoni, S., Å. T. Røe, …, N. P. Smith. 2016. The calcium-frequency response in the rat ventricular myocyte: an experimental and modelling study. *J. Physiol.* 594:4193–4224.

50. Healy, L. J., Y. Jiang, and E. W. Hsu. 2011. Quantitative comparison of myocardial fiber structure between mice, rabbit, and sheep using diffusion tensor cardiovascular magnetic resonance. *J. Cardiovasc. Magn. Reson.* 13:74.

51. Bishop, M. J., and G. Plank. 2012. The role of fine-scale anatomical structure in the dynamics of reentry in computational models of the rabbit ventricles. *J. Physiol.* 590:4515–4535.

52. Bayer, J., A. J. Prassl, …, E. J. Vigmond. 2018. Universal ventricular coordinates: a generic framework for describing position within the heart and transferring data. *Med. Image Anal.* 45:83–93.

53. Beeler, G. W., and H. Reuter. 1977. Reconstruction of the action potential of ventricular myocardial fibres. *J. Physiol.* 268:177–210.

Biophysical Journal, Volume *117*

Supplemental Information

Investigation of the Role of Myocyte Orientations in Cardiac Arrhythmia Using Image-Based Models

Dominic G. Whittaker, Alan P. Benson, Irvin Teh, Jürgen E. Schneider, and Michael A. Colman

1. Supplementary Figures

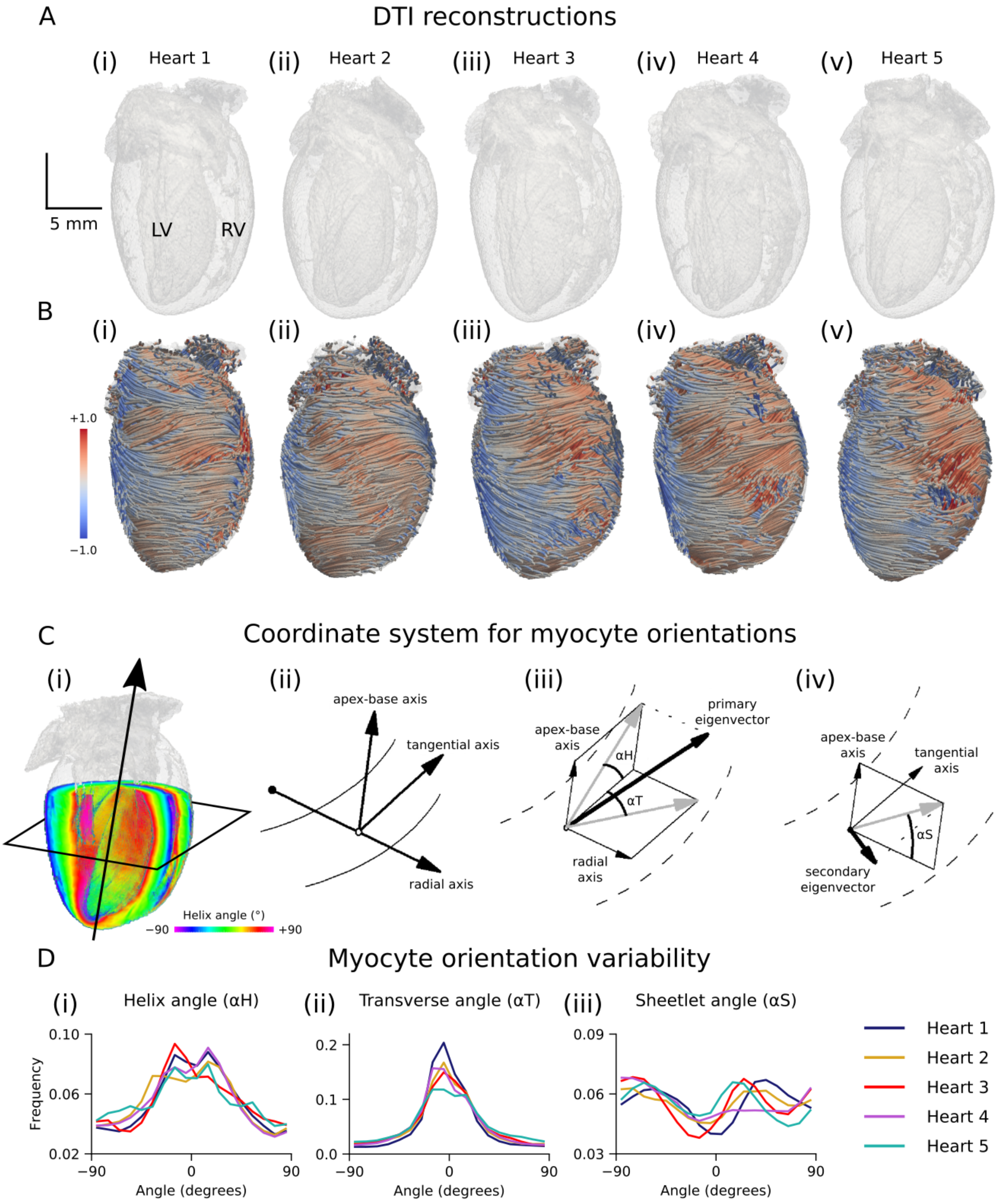

Figure S1: Anatomical variability from DTI reconstructions and co-ordinate system used to quantify myocyte organisation. (A) Five healthy rat heart reconstructions from *ex vivo* DTI (1), and (B) streamlines coloured according to the *z* component of the myocyte orientation. (C) Co-ordinate system used to compute myocyte and sheetlet orientation angles.

(i) For each heart a base-apex axis is fitted to the centre of the left ventricle, normal to the transverse plane of the heart (shown as a rectangle in the short-axis plane). (ii) Three orthogonal reference axes are defined for each voxel, from which (iii) the helix angle (αH) and transverse angle (αT) are calculated from the primary eigenvector, and (iv) sheetlet angle (αS) is calculated from the secondary eigenvector (2). (D) Myocyte orientation variability between the five ventricular geometries – (i) helix angle, (ii) transverse angle, and (iii) sheetlet angle.

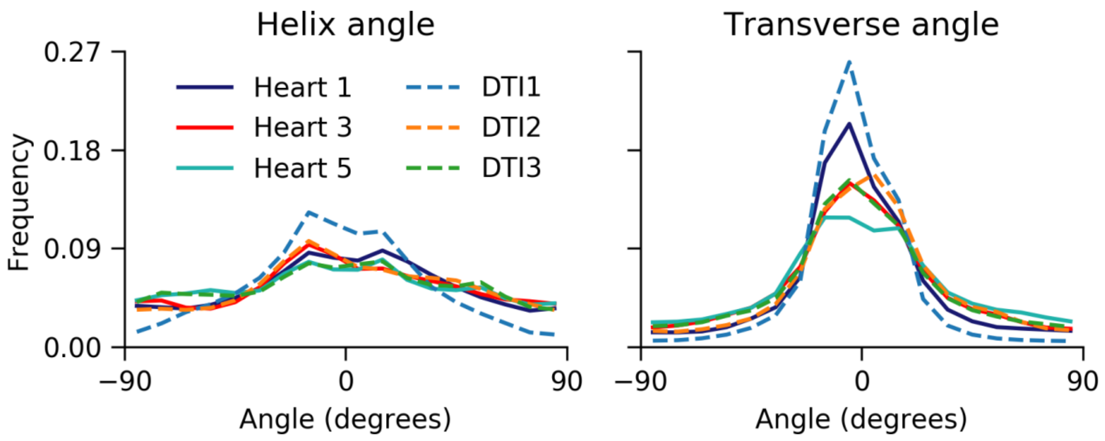

Figure S2: Myocyte orientation variability in the processed DTI datasets. Variability between the three DTI-based myocyte orientation scenarios as quantified by the helix angle (left panel) and transverse angle (right panel), compared with the unprocessed DTI data from Hearts 1, 3, and 5.

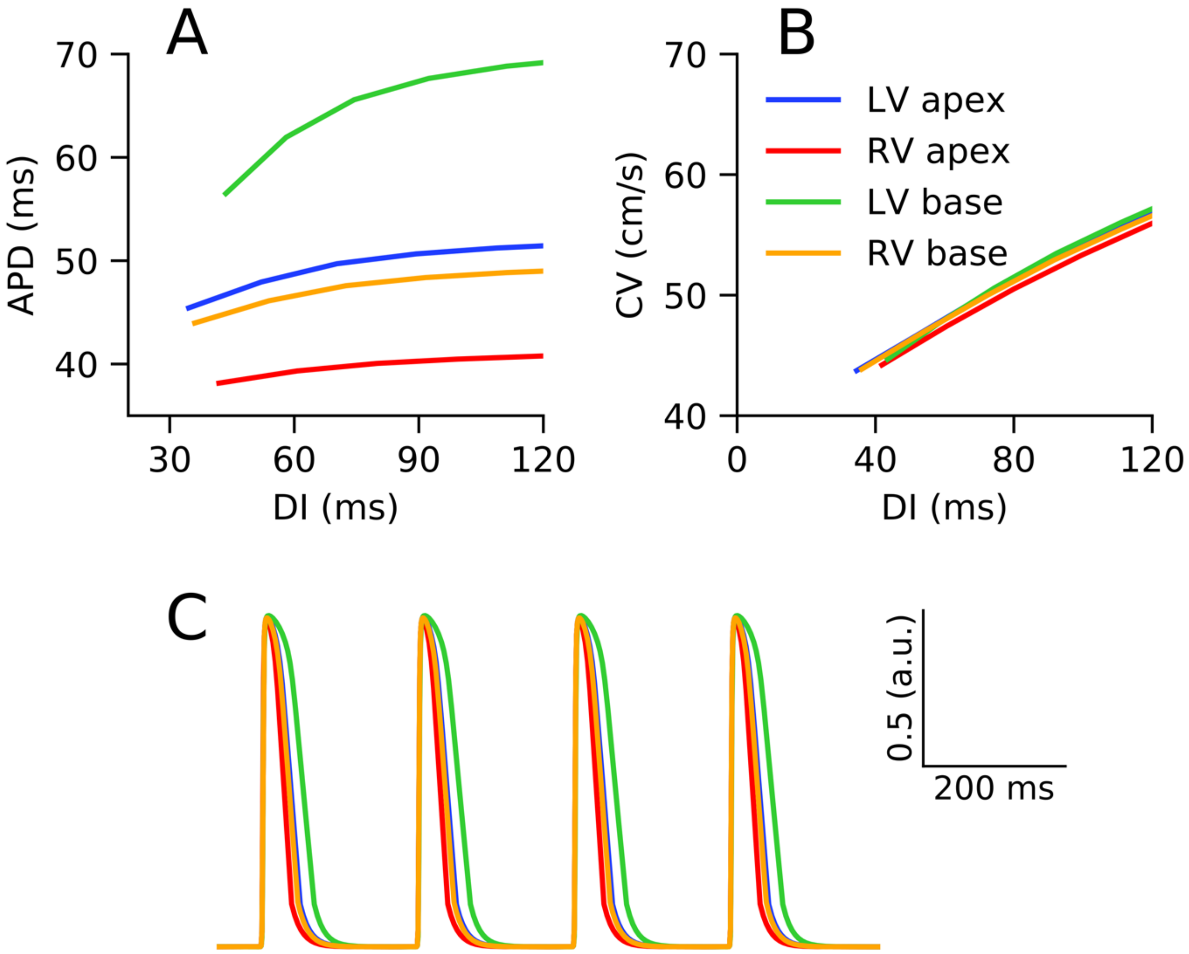

Figure S3: Electrophysiological heterogeneity in Protocol 2. (A) Action potential duration and (B) conduction velocity restitution curves for different regions of the ventricles, used in Protocol 2. (C) A train of rat ventricular cell action potentials using the modified FK3V regional cell models at a pacing rate of 5 Hz. Regional cell models were created by adjusting the τ_r parameter in the updated FK3V model by ±20% in order to reproduce experimental differences in rat ventricular APD (3).

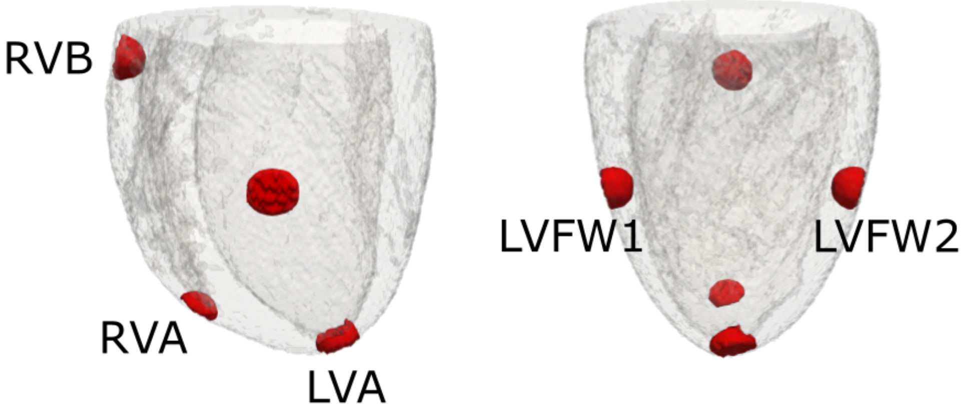

Figure S4: Stimulus locations for Protocol 2. The bi-ventricular geometry used in this study (shown in transparent grey), with stimulus locations used in Protocol 2 highlighted in red. Locations are as follows: right ventricular base (RVB); right and left ventricular apex (RVA and LVA, respectively); left ventricular free wall location 1 and 2 (LVFW1 and LVFW2, respectively).

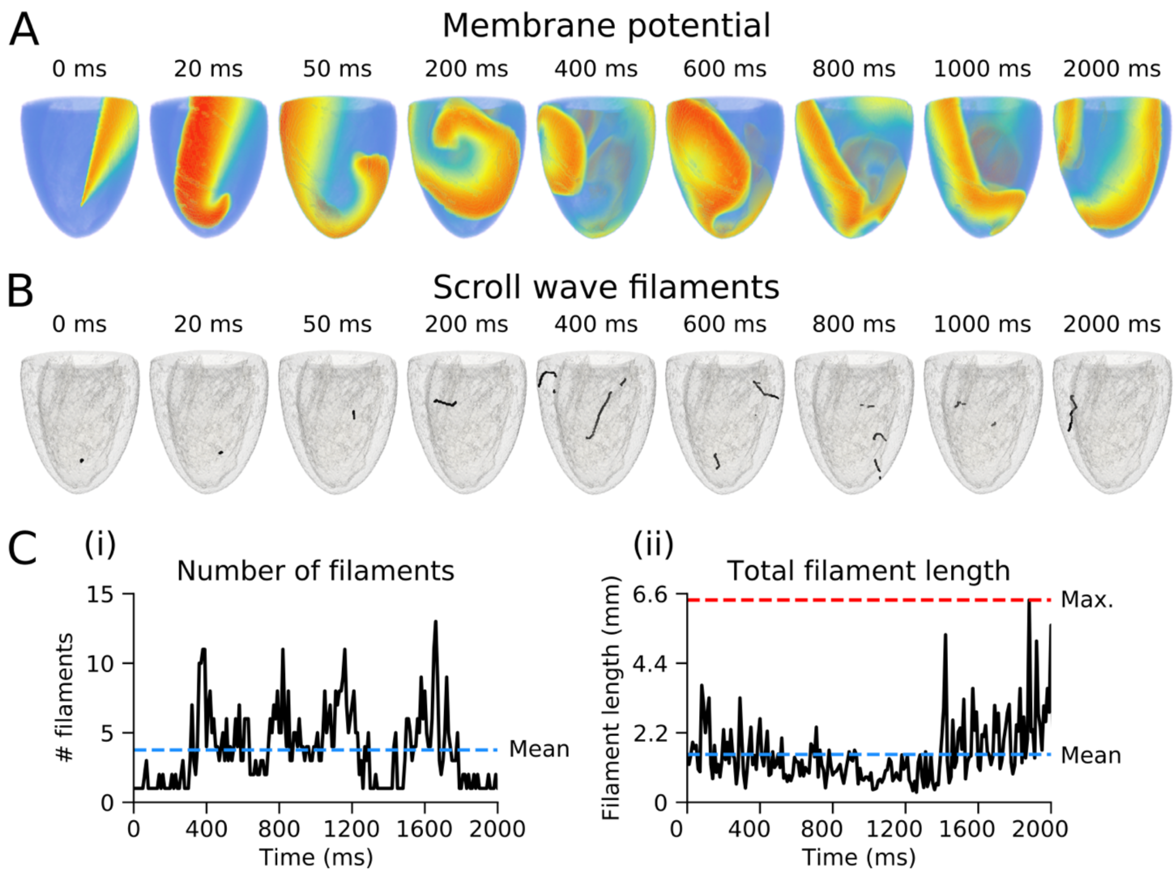

Figure S5: Arrhythmia initiation and scroll wave filaments. (A) Initiation and evolution of scroll waves on the left ventricular lateral wall using the phase distribution method (4). (B) Corresponding scroll wave filaments shown as black lines on a semi-transparent contour of the geometry. (C) Time series of (i) the number of filaments over a 2000 ms period (mean is shown with a blue dotted line), and (ii) the total filament length (mean and max. shown with blue and red dotted lines, respectively).

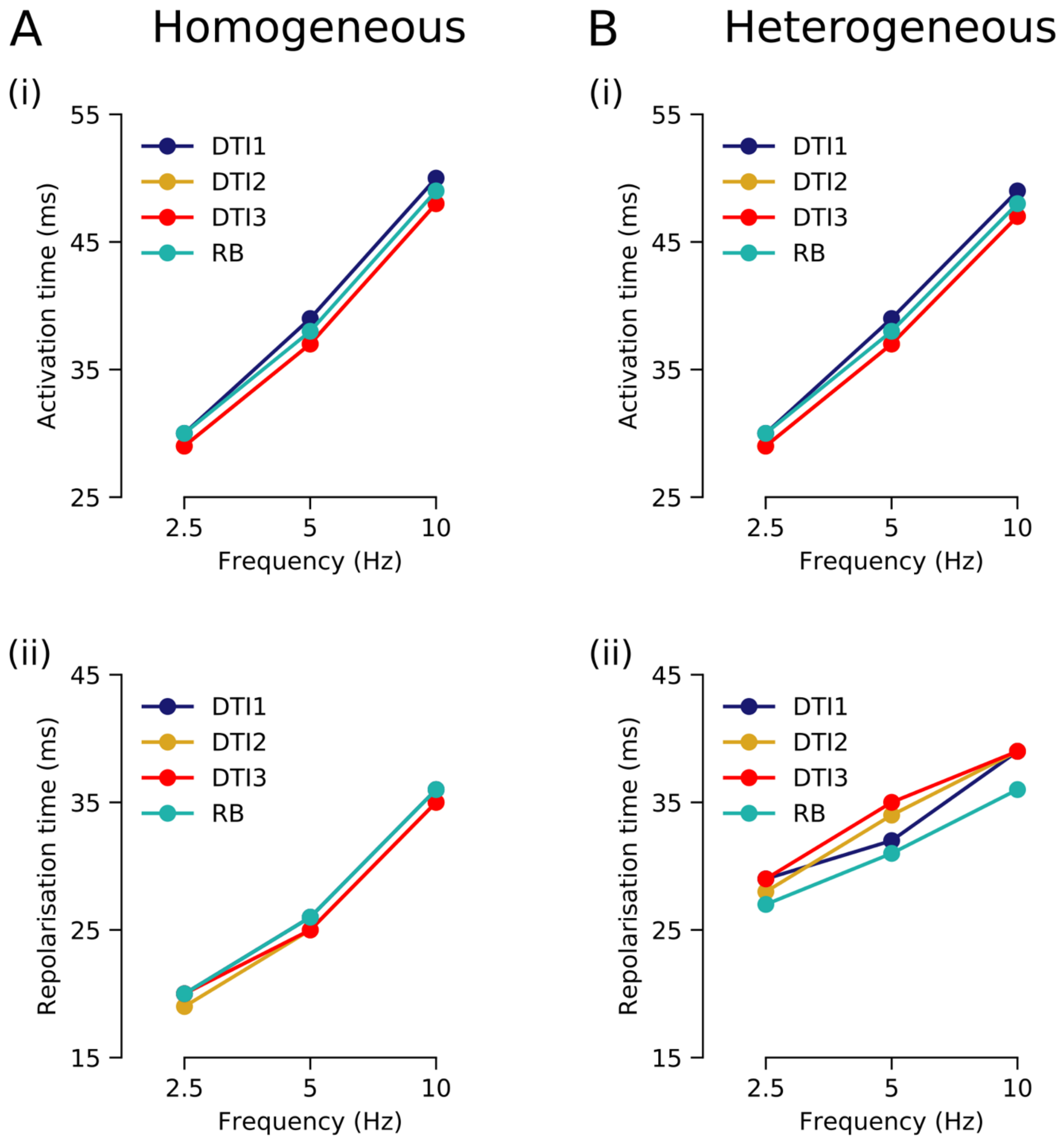

Figure S6: Effects of myocyte orientations on activation and repolarisation times. A summary of total (i) activation and (ii) repolarisation times for all myocyte orientation scenarios under (A) homogeneous and (B) heterogeneous conditions at pacing rates of 2.5, 5, and 10 Hz (corresponding to cycle lengths of 400, 200, and 100 ms, respectively).

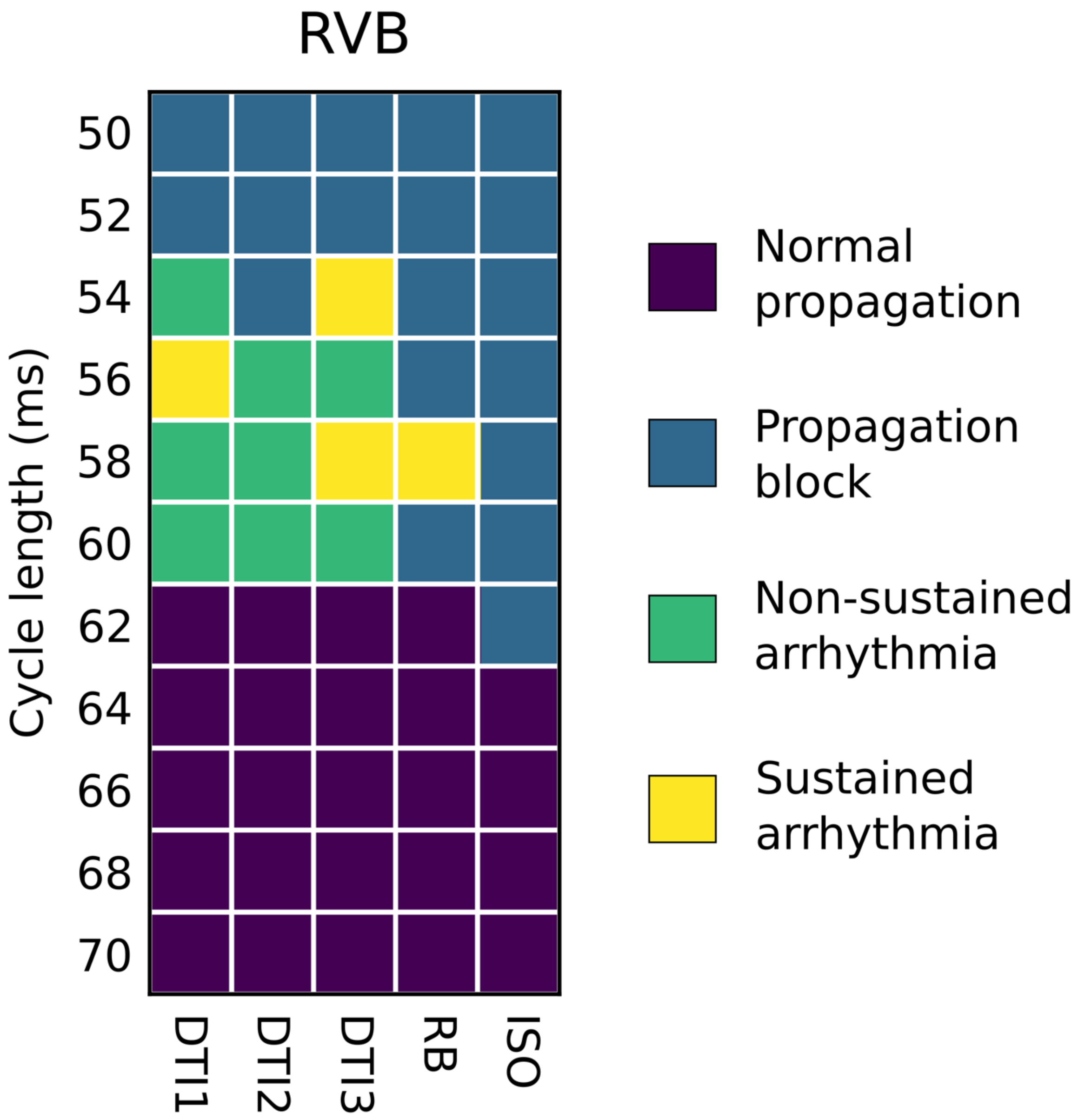

Figure S7: Comparison of arrhythmia inducibility under anisotropic and isotropic conditions. Vulnerability grids for arrhythmia inducibility following rapid pacing across a range of cycle lengths at the right ventricular base (RVB) under the four myocyte orientation scenarios and under isotropic (ISO) conditions.

3. Supplementary Tables

Supplementary Table S1: A summary of data from (1, 5) used in this study.

	Heart 1	Heart 2	Heart 3	Heart 4	Heart 5
**Total volume (mL)**	0.556	0.627	0.546	0.609	0.553
**Myocyte orientations in this study**	DTI1		DTI2		DTI3

A summary of the data used to create the hybrid bi-ventricular geometry and DTI-based myocyte orientation scenarios used in this study. Healthy rat heart reconstructions from DTI at 100 μm resolution (1) were previously cropped into bi-ventricular geometries (5), with the total volumes shown in the table. The three geometries with the closest total volumes (1, 3, and 5) were merged to form a single bi-ventricular geometry with the microstructure from Hearts 1, 3, and 5 forming the basis of myocyte orientation scenarios DTI1, DTI2, and DTI3, respectively.

Supplementary Table S2: A summary of arrhythmia inducibility for Protocol 2.

	**RV apex**	**RVB**	**LVFW1**	**LVFW2**	**LV apex**	**Total**
**DTI1**	× (0)	✓ (4)	✓ (1)	× (0)	× (0)	5
**DTI2**	✓ (2)	✓ (3)	✓ (1)	× (0)	✓ (3)	9
**DTI3**	✓ (1)	✓ (4)	✓ (1)	× (0)	✓ (2)	8
**RB**	✓ (1)	✓ (1)	✓ (2)	✓ (1)	✓ (1)	6

A summary of whether or not an arrhythmia was inducible for DTI-based (DTI1, DTI2, and DTI3) and rule-based (RB) myocyte orientations for five locations – right ventricular (RV) apex, RV base, left ventricular free wall 1 and 2 (LVFW1 and LVFW2, respectively), and LV apex, as well as a total across all five locations. Boxes marked with a cross denote that no arrhythmias were inducible, whereas boxes marked with a tick denote that an arrhythmia was inducible (the number of pacing rates tested at which re-entry could be induced is given in brackets).

Supplementary Table S3: *p* values for one-way ANOVA.

	***p* value**
**Mean number of filaments**	0.569
**Mean total filament length**	0.395
**Max. total filament length**	0.521

A summary of *p* values from one-way ANOVA for the mean number of filaments, mean total filament length, and maximum total filament length from $n = 10$ re-entry simulations.

4. Supplementary Videos

Video S1: Effects of myocyte orientation variability on ventricular activation. A cross-sectional view into the left and right ventricular cavities showing activation over a 100 ms period under different myocyte orientation scenarios at a pacing rate of 5 Hz. Excited tissue is red whereas recovered tissue is blue.

Video S2: Effects of myocyte orientation variability on arrhythmia induction. A right ventricular anterior wall view of rapid pacing at a cycle length of 60 ms at the right ventricular apex under different myocyte orientation scenarios, shown over a period of 1000 ms. Excited tissue is red whereas recovered tissue is blue.

Video S3: Effects of myocyte orientation variability on arrhythmia dynamics. A left ventricular anterior wall view of the dynamic evolution of re-entrant waves and corresponding scroll wave filaments over a 1000 ms period. Filaments are shown as black lines on a semi-transparent contour of the geometry. Excited tissue is red whereas recovered tissue is blue.

Video S4: A mechanism of re-entry induction in Protocol 2 due to myocyte organisation. A bi-ventricular wedge view of wave propagation (excited tissue shown in red on a semi-transparent contour of the geometry) and myocyte orientation streamlines for the DTI1 (left) and DTI2 (right) scenarios, following left ventricular free wall stimulation (LVFW1) in Protocol 2. For DTI2 the initial stimulus wave propagates preferentially along the vertical axis compared to more horizontally for DTI1. The second stimulus captures and travels towards the RV (a region of lower APD) where wave breakthrough occurs for DTI1, whereas

for DTI2 the wave travels more along the direction of apico-basal heterogeneity, and so meets the longer APD of the base. In both cases the APD is longer in the LV than RV, so the wave gets blocked as it tries to propagates away from the RV (to the right in the video). This critical difference leads to the fourth and fifth (but not third) stimuli being captured for DTI1, compared to the third and fifth stimuli for DTI2. The repolarisation heterogeneity induced by multiple rapid activations causes the fifth stimulus to generate a wave which breaks for DTI1, causing even more repolarisation asymmetry. This ultimately causes the wave from the sixth stimulus to break and degenerate into re-entry for DTI1, but not DTI2.

References

1. Teh, I., D. McClymont, R.A.B. Burton, M.L. Maguire, H.J. Whittington, C.A. Lygate, P. Kohl, and J.E. Schneider. 2016. Resolving Fine Cardiac Structures in Rats with High-Resolution Diffusion Tensor Imaging. Sci. Rep. 6: 30573.

2. Benson, A.P., O. Bernus, H. Dierckx, S.H. Gilbert, J.P. Greenwood, A.V. Holden, K. Mohee, S. Plein, A. Radjenovic, M.E. Ries, G.L. Smith, S. Sourbron, and R.D. Walton. 2011. Construction and validation of anisotropic and orthotropic ventricular geometries for quantitative predictive cardiac electrophysiology. Interface Focus. 1: 101–116.

3. Watanabe, T., L.M.D. Delbridge, J.O. Bustamante, and T.F. Mcdonald. 1983. Heterogeneity of the action potential in isolated rat ventricular myocytes and tissue. Circ. Res. 52: 280–290.

4. Biktashev, V.N., and A.V. Holden. 1998. Reentrant waves and their elimination in a model of mammalian ventricular tissue. Chaos Interdiscip. J. Nonlinear Sci. 8: 48–56.

5. Whittaker, D.G., A.P. Benson, I. Teh, J.E. Schneider, and M.A. Colman. 2018. Role of cardiac microstructure variability on ventricular arrhythmogenesis. In: 2018 Computing in Cardiology Conference (CinC). .
